# Development of an Accurate and Sensitive Diagnostic System Based on Conventional PCR for Detection of African Swine Fever Virus in Food Waste

**DOI:** 10.1007/s12088-022-01007-y

**Published:** 2022-03-18

**Authors:** Siwon Lee, Junhwa Kwon, Bo-Young Kim, Jin-Ho Kim

**Affiliations:** 1 R&D Team, LSLK Co. Ltd., Gimpo, 10111 Republic of Korea; 2 Waste-To-Energy Research Division, Environmental Resources Research Department, National Institute of Environmental Research, Incheon, 22689 Republic of Korea; 3 Department of Chemical Engineering & Biotechnology, Tech University of Korea, Siheung, Gyeonggi-do 15073 Republic of Korea; 4 Institute of Tissue Regeneration Engineering (ITREN), Dankook University, Cheonan-si, Chungcheongnam-do 31116 Republic of Korea; 5 Department of Chemistry, College of Science and Engineering, Dankook University, Cheonan-si, Chungcheongnam-do 31116 Republic of Korea

**Keywords:** African swine fever virus (ASFV), ASFV-modified positive material, ASFV monitoring, Food waste, Genotyping

## Abstract

*African swine fever virus* (ASFV), a highly contagious virus, can cause diseases with high mortality rates in pigs, making it a pathogen of social and economic significance. ASFV has been reported to show potential long-term survival in living livestock, such as pigs, but also in leftover cooking meat and undercooked pork meat. Hence, it is possible that there could be direct reinfection or secondary infection through feed produced from household food waste and treatment facilities. Many polymerase chain reaction (PCR)-based molecular diagnostic techniques to detect ASFV in clinical swine samples have been reported. However, those with applicability for food waste samples, which contain relatively low viral copy numbers and may contain various unknown inhibitors of PCR, are still lacking. In this study, we developed a conventional PCR-based diagnostic system that can detect ASFV with high sensitivity from food waste sample types. The technique shows a 10–100 times higher limit of detection compared to that of previously reported methods based on conventional PCR and quantitative real-time PCR. It is also capable of amplifying a sequence that is approximately 751 nucleotides, which is advantageous for similarity analysis and genotyping. Moreover, a ASFV-modified positive material different from ASFV that could synthesize 1400 nucleotide amplicons was developed to identify false-positive cases and thus enhance diagnostic accuracy. The method developed herein may be applicable for future ASFV monitoring, identification, and genotyping in food waste samples.

**Supplementary Information:**

The online version contains supplementary material available at 10.1007/s12088-022-01007-y.

## Introduction

*African swine fever virus* (ASFV) is a highly contagious virus that infects wild and domestic pigs. Diseases caused by ASFV have a substantially high mortality rate [1]. Vaccines and treatments for ASFV have not yet been developed and consequently, if ASFV is detected in any country, it must be immediately reported to the World Organization for Animal Health (OIE), and the country may be required to take measures, such as halting all pig-related international trade. This creates a threat for the pork industry and the stability of food supply, making ASFV a socially and economically significant pathogen [2]. Previous research on ASFV has covered topics such as its evolution and taxonomy, diagnosis, outbreaks, epidemiology, vaccines, and control. Diagnostic research has specifically focused on the monitoring of clinical swine samples [3–9]. However, as the potential for the long-term survival of ASFV in leftover meat and undercooked pork meat has now been recognized [10], the risk of secondary infection from household or treatment facility food waste and livestock feed reproduced from such food waste has been acknowledged [1, 2]. The African swine fever risk reminder from England recommends not feeding kitchen or catering waste to pigs. South Korea has also taken measures such as the amendment to the Enforcement Decree of the Waste Control Act to limit the feeding of food waste after direct treatments in pig farms, and the limitation of food waste-based feed for pigs. Preemptive monitoring of ASFV in food wastes from households, cafeterias, animal feed, and manure treatment facilities remains warranted [2, 11]. ASFV is generally diagnosed using polymerase chain reaction (PCR)-based molecular methods, but these techniques have been primarily developed to monitor clinical swine samples [6, 9, 12–14]. However, a relatively low viral copy number is expected in environmental samples, such as food waste, compared to that in clinical pig samples. Furthermore, a high level of sensitivity is required to detect pathogens from the unknown PCR inhibitors included in the total DNA, but to the best of our knowledge, no previous study has compared the different molecular diagnostic technologies for monitoring ASFV in food waste. Most recently, quantitative real-time PCR (qPCR) methods with various reagents (such as SYBR Green, lyophilized powder, and/or endogenous internal control) or newly targeted genes (A137R, B646L [p72], MGF360-505R, and/or CD2v genes) for the detection of ASFV have been developed [15–18]. However, conventional PCR is still used frequently, because it can amplify relatively longer DNA fragments when compared to qPCR and the amplicons can be used for sequencing to allow for follow-up similarity and genotyping analyses. Moreover, PCR primers and *Taq* polymerase are inexpensive and have a large user base, which make them appropriate for standard test methods [19]. Therefore, the Korea Ministry of Agriculture, Food and Rural Affairs and the Ministry of Environment both use conventional PCR techniques with qPCR for the diagnosis of ASFV. To ensure their accuracy and reliability, PCR tests require positive controls. In general, for molecular biology-based tests, a device to check for positive control contamination must be included [19, 20]. The positive controls used in the conventional PCR-based ASFV detection methods however, are based on synthesized plasmid DNA and consequently, the partial sequences of some genes and the false-positives caused by the positive control cannot be identified. This study aimed to develop a diagnostic system that can detect ASFV in food waste samples with a high sensitivity and allow for the results to be compared with previously reported PCR primer sets for ASFV detection and performance in samples. In addition, an ASFV-modified positive material has been developed to detect false positives, thereby creating a diagnostic system that can detect ASFV-specific genes in food waste samples.

## Material and Methods

### Design of Specific PCR Primers and Nucleic Acid Collection

To design ASFV-specific primers, 54 ASFV isolates, including ASFV isolate HBNH-2019 (National Center for Biotechnology Information [NCBI] accession number MN207061.1), reference viruses (*porcine circovirus* 2 [PCV2; NC_005148], *porcine parvovirus* [PPV; NC_001718], and *pseudorabies virus* [PrV; NC_006151]) sequences were collected from the NCBI. The DNAMAN software package (version 6.0; Lynnon Biosoft, Quebec, Canada) was used to select the primers with 51–59 °C (optimum, 55 °C) T_m_ values from the ASFV isolate HBNH-2019 P72–73 partial gene sequence. The candidate PCR primers that did not show potential hairpin structures and self-annealing in Oligo Calculator version 3.27 were selected [19, 21]. BioEdit version 7.2.6 was used for multiple sequence alignment of the candidate PCR primers with collected reference sequences. The candidate PCR primers were modified using the nucleotide letter code to be compatible with the sequences of the 54 ASFV isolates (Table 1). The ASFV P72–73 gene partial sequence (1,941 nucleotides [nt]), PCV2 (NC_005148 [1034–1283, and 250 nt]), PPV (NC_001718 [2387–2636, and 250 nt]), and PrV (NC_006151 [66,781–67,030, and 250 nt]) were synthesized by Marcrogen Co. Ltd. (Seoul, Korea) and inserted into the pUC57 vector that has the restriction sites of *Eco*RI (5′-GAATTC-3′) and *Hind*III (5′-AAGCTT-3′).

**Table 1:** Development and reference PCR primer sets for the detection of *African swine fever virus*

Division	Primer name		Sequence (5′→3′)	Length (nt)	Location^**a**^		Product size (nt)	PCR condition (Temp., time)			Reference
					Start	End		1st DN	DN, AN & EX^c^	FE	
Development PCR primers	*Forward*										
	ASFV_157F		GAAGAAACACATWTGGTGC	19	157	175	300–800^b^	95 °C, 5 min	95 °C (30 s), 55 °C (30 s) & 72 °C (60 s) (35 cycles)	72 °C, 5 min	This study
	ASFV_408F		GTTTCCTCGCAACGGATA	18	408	425					
	ASFV_634F		CGCAAATTTTGCATCCCA	18	634	651					
	ASFV_783F		AAATGATACGCAGCGAAC	18	783	800					
	ASFV_806F		GCCATACCAACCCGAAAT	18	806	823					
	ASFV_861F		CCAAACAGCAGGTAAACA	18	861	878					
	ASFV_1030F		GCTATTCCCTCRGTRTCCAT	20	1,030	1,049					
	ASFV_1160F		GTAGACGCAATATACGCTTTA	21	1,160	1,180					
	*Reverse*										
	ASFV_811R		TATGGCTACACGTTCGCT	18	794	811					
	ASFV_817R		CRYAGCCATACCAACC	16	802	817					
	ASFV_1020R		GTTCTCATTAAACCAAAAGCG	21	1,000	1,020					
	ASFV_1236R		CGCTCACGAATAATGAACTT	20	1,217	1,236					
	ASFV_1354R		CSAAYAATAACCAYCACG	18	1,337	1,354					
	ASFV_1384R		GCCATTTAAGAGCAGACATT	20	1,365	1,384					
	ASFV_1434R		ACCTGGAAYATYTCCGA	17	1,418	1,434					
	ASFV_1517R		YACYCACCACGCAGARRT	18	1,500	1,517					
Reference PCR primer sets	#1	PPA-1	AGTTATGGGAAACCCGACCC	20	360	379	257	95 °C, 10 min	95 °C (15 s), 62 °C (30 s) & 72 °C (30 s) (40 cycles)	72 °C, 7 min	[2]
		PPA-2	CCCTGAATCGGAGCATCCT	19	598	616					
	#2	P72-U	GGCACAAGTTCGGACATGT	19	1,460	1,478	479	95 °C, 10 min	95 °C (30 s), 48 °C (30 s) & 72 °C (60 s) (40 cycles)	72 °C, 10 min	[6, 9]
		P72-D	GTACTGTAACGCAGCACAG	19	1,919	1,938					
	#3	F	GTCTTATTGCTAACGATGGGAAG	23	23	45	256	95 °C, 10 min	95 °C (30 s), 54 °C (30 s) & 72 °C (60 s) (40 cycles)	72 °C, 10 min	[14]
		R	CCAAAGGTAAGCTTGTTTCCCAA	23	256	278					
	#4	72ARs	GACGCAACGTATCTGGACAT	20	897	917	310	95 °C, 7 min	95 °C (30 s), 60 °C (30 s) & 72 °C (30 s) (35 cycles)	72 °C, 10 min	[13]
		72ARas	TTTCAGGGGTTACAAACAGG	20	1,185	1,206					
	#5	72Ns	TACTATCAGCCCCCTCTTGC	20	964	983	243	95 °C, 7 min	95 °C (30 s), 61 °C (30 s) & 72 °C (30 s) (35 cycles)	72 °C, 7 min	
		72Nas	AATGACTCCTGGGATAAACCAT	22	1,248	1,206					

^a^Based on NCBI accession number MK554698.1

^b^PCR primer sets that formed amplification products in the range of 400–800 nt were included

^c^DN, AN & EX are Denaturation, Annealing & Extension, respectively

### Selection of ASFV-Specific PCR Primer Sets

Candidate PCR primer sets were combined and expected to produce amplicons 300–800 nt in length. The specific reactions of the candidate PCR primer sets were confirmed using the ASFV plasmid 10^–3^ (1 pg/μL). Candidate PCR primer sets showed the expected PCR product sizes (400–800 nt) based on the amplification strength and region of amplification, which were tested with the three reference viruses. If products showed non-specific reactions or had low reproducibility for positive reactions, they were excluded. A sensitivity test was performed on selected PCR primer sets that demonstrated specific reactions to ASFV. The ASFV plasmid (1 ng/μL) was diluted ten-fold to 10^–9^ (1 ag/μL), and 10^–4^–10^–9^ was used for the sensitivity test. For the PCR primer sets that showed the most outstanding detection sensitivity, amplification strength for the sensitivity of end-point and the size of the PCR products were analyzed to select the final PCR primer set. The PCR mixture was as follows: 10 μL of AccuPower® HotStart PCR PreMix (Bioneer, Korea), 2 μL of candidate PCR primer (forward and reverse primer [25 pmol 1 μL, each]), 7 μL of nucleic acid-free water, and 1 μL of template. PCR conditions were as follows: denaturation (95 °C, 5 min); 35 cycles of 95 °C for 30 s, 55 °C for 30 s, and 72 °C for 60 s; and a final extension (72 °C, 5 min).

### Sample and Artificial Infection Test

Twenty pork meat-based food waste samples were collected from intermediate processing, biogas, and composting treatment facilities and stored in a deep freezer in the laboratory. For each sample, 1 g of sample and 7 mL of phosphate buffered saline were mixed with 15 mL of the Homogenize Kit buffer (Innogenetech, Korea), and ground for 40 s at 6 m/s using the MP FastPrep® 24 (MP Biomedicals, USA) [21]. Total DNA was extracted from 140 μL of the ground solution using the QIAamp® DNA Mini kit (Qiagen, Germany), according to the manufacturer’s instructions. The concentration and purity of extracted DNA was examined using a DS-11 Spectrophotometer (DeNOVIX, USA). The developed ASFV PCR primer set was used to detect ASFV in the extracted DNA. Moreover, when all samples were found to be negative, artificial infection and sensitivity were analyzed to test the performance of the ASFV PCR primer sets and to verify the effects of unknown PCR inhibitors. DNA (600 μL) was extracted from one pork sample using the same extraction kit and method and used as a dilution solvent. Then, 45 μL of the dilution solvent and 5 μL of the ASFV plasmid (1 ng/μL) were mixed to form 10^–1^ artificially infected DNA, which was ten-fold serially diluted to 10^–8^ (10 ag/μL). The artificially infected DNA was used to analyze the performance and sensitivity of the developed PCR primer sets for ASFV detection in the samples.

### Comparison of Reference PCR Primer Sets for the Detection of ASFV

Weber et al. [6] conducted a comparative experiment on five conventional PCR primer sets that could detect ASFV (Table 1). Specific reactions with the ASFV plasmid, non-specific reactions with the three reference viruses, detection of ASFV in 20 DNA samples from food waste samples, and sensitivity from artificial infections were all analyzed. All templates were identical to those used in the test for the developed PCR primer sets, and AccuPower® HotStart PCR PreMix (Bioneer) and SimpliAmp Thermal Cycler (Applied Biosystems, USA) were used. The PCR conditions used were as previously described [6, 9, 12–14].

### Design and Test of Positive Control Equipped with False-Positive Checking Device

To identify the false positives caused by contamination from the positive control and enhance the accuracy of the diagnostic test, a positive control was designed with a modified sequence. *Porcine epidemic diarrhea virus* (PEDV; NC_003436.1:26,374–27,699; 1,326 nt) sequences were inserted such that the conventional PCR primer set developed in this study for ASFV detection could amplify products of different sizes; the *Eco*RV restriction enzyme site (5′-CATATC-3′) was included in the PEDV sequence for treatment of the PCR product with *Eco*RV (Enzynomics, Korea). The reaction of the developed PCR primer set with ASFV-modified positive control would produce PCR products of approximately 1,400 nt, which is different in size than ASFV. When this PCR product is treated with the *Eco*RV for digestion, it produces two bands (500 + 900 nt in size). Conditions of restriction enzyme treatment were as follows: 5 μL of the PCR product, 2 μL of 10X EzBuffer III, 1 μL of *Eco*RV (Enzynomics, Korea), and 12 μL of sterile water were mixed and incubated at 37 °C for 1 h. The reaction product was mixed with 6X loading dye and electrophoresed on a 2% agarose gel. The results were examined using a gel documentation system.

### Validation

The developed PCR method was validated by focusing on reproducibility: three different researchers conducted the experiment on 3 different days, according to the 2010 report of the National Institute of Food and Drug Safety Evaluation in Republic of Korea [22]. A sensitivity test was conducted using samples of ASFV plasmid 10^–3^ (1 pg/μL)–10^–8^ (10 ag/μL) with artificial infections, and an analytical quality control (AQC) test was performed using 10 samples (four positives [ASFV concentration of 100 fg/μL, 10 fg/μL, 1 fg/μL, and 100 ag/μL] and six negatives) that were randomly produced.

## Results and Discussion

### Design of Specific PCR Primers and the Selection of an ASFV Specific PCR Primer Set

**Fig. 1:**
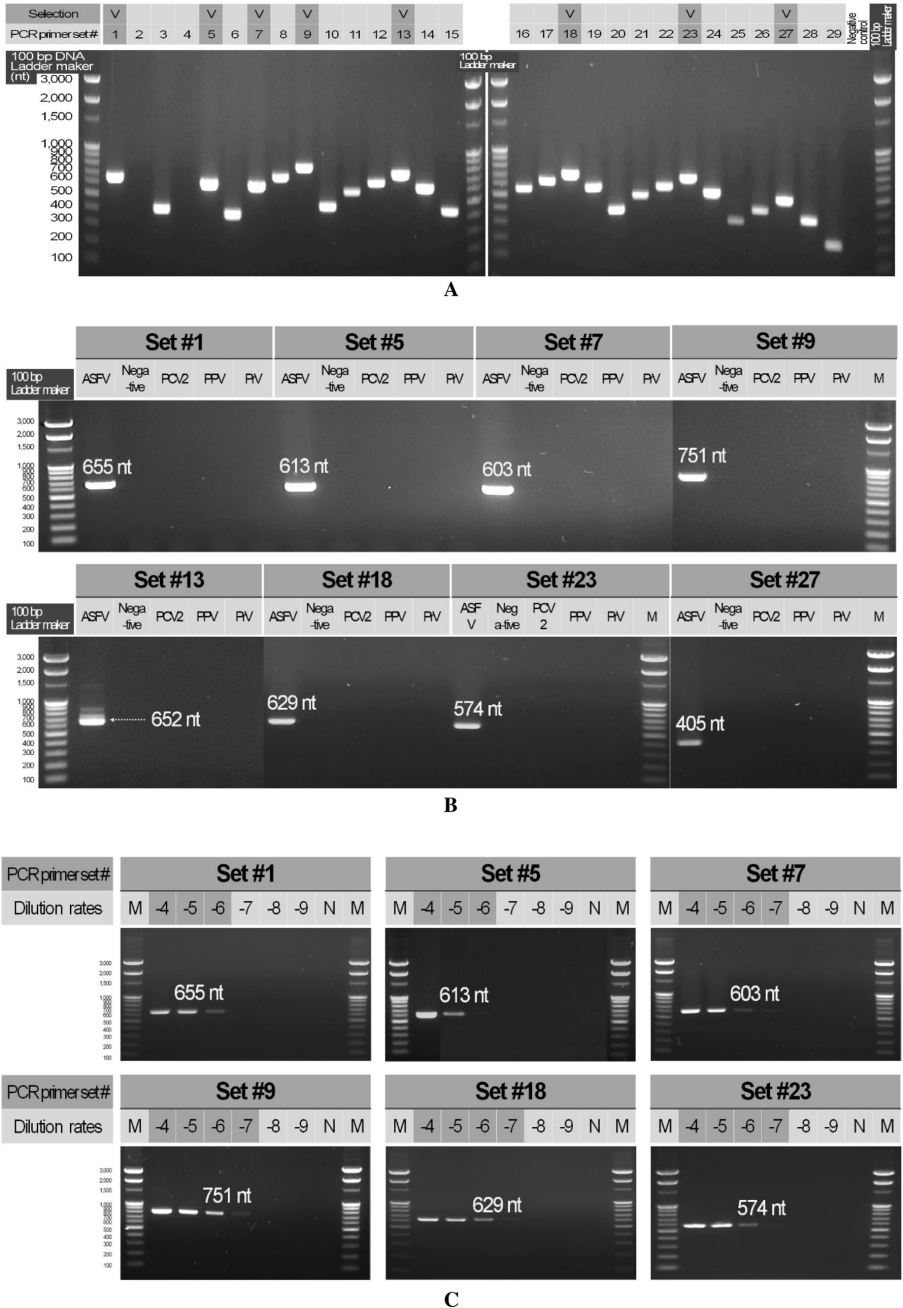
Specific, non-specific, and sensitivity tests for the PCR primer sets developed for the detection of *African swine fever virus.* 100 bp DNA ladder marker (Enzynomics, Korea). Panel A: Specific reactions; V, selected primer sets. Panel B: Non-specific reactions of the reference viruses. ASFV, *African swine fever virus*; PCV2, *porcine circovirus* 2; PPV, *porcine parvovirus*; PrV, *pseudorabies virus* Panel C: Sensitivity test –4 to –9, dilution value for the template from ASFV plasmid concentration of 1 ng/μL; N, negative control

**Fig. 2:**
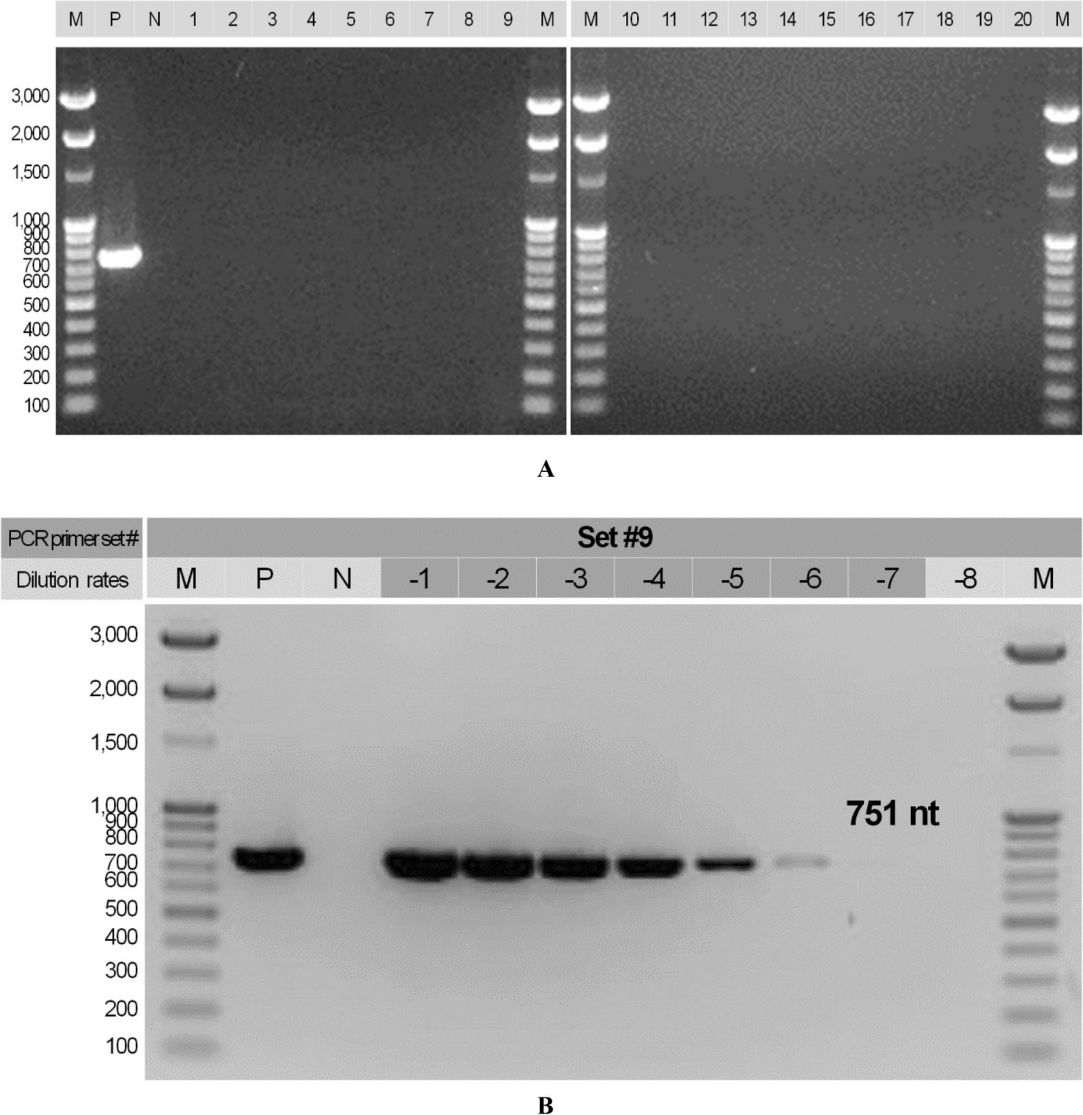
*African swine fever virus* (ASFV) monitoring in twenty food waste samples (panel A) and sensitivity tests using ASFV artificial infections (panel B) M, 100 bp DNA ladder marker (Enzynomics); P, positive control; N, negative control; 1–20, number of food waste samples; –1 to –8; concentrations of ASFV plasmid in the artificial infection samples

Sixteen candidate PCR primers (eight forward and eight reverse) that were expected to show specific annealing to ASFV DNA were constructed. In total, 29 candidate PCR primer sets were combined and were expected to produce 300–800 nt amplicons from the ASFV DNA (Table 1). In the results of the specificity test, 28 of the 29 combinations showed the expected band sizes, and eight candidate PCR primer sets were selected based on amplification strength, product size, and region of amplification (Fig. 1A). No non-specific reactions were found in the eight candidate PCR primer sets for PCV2, PPV2, and PrV. However, different-sized bands and weak amplicons were found in the positive reaction with ASFV for PCR primer set #13 and #27, respectively; therefore, they were excluded from further experiments (Fig. 1B). As a result of the sensitivity analysis for the six candidate PCR primer sets that were confirmed for specific and non-specific reactions, the sensitivity of PCR primer sets #1, #5, and #18 was 10^–6^ (1 fg/μL), while that for #7, #9, and #23 was 10^–7^ (100 ag/μL). Of these, PCR primer set #9 (ASFV_634F and ASFV_1384R, 751 nt) was selected because of its amplicon size and outstanding reaction strength at 10^–7^ (100 ag/μL; Fig. 1C). To confirm the applicability of PCR primer set #9 for food waste samples, the ASFV in 20 total DNA samples was analyzed, and all results were negative (Fig. 2A). Artificial infection tests were consequently conducted, demonstrating the sensitivity to be 10^–7^ (100 ag/μL) for the total DNA extracted from the sample (Fig. 2B). Therefore, it was validated for application in food waste samples.

### Comparison of Reference PCR Primer Sets for the Detection of ASFV

**Fig. 3:**
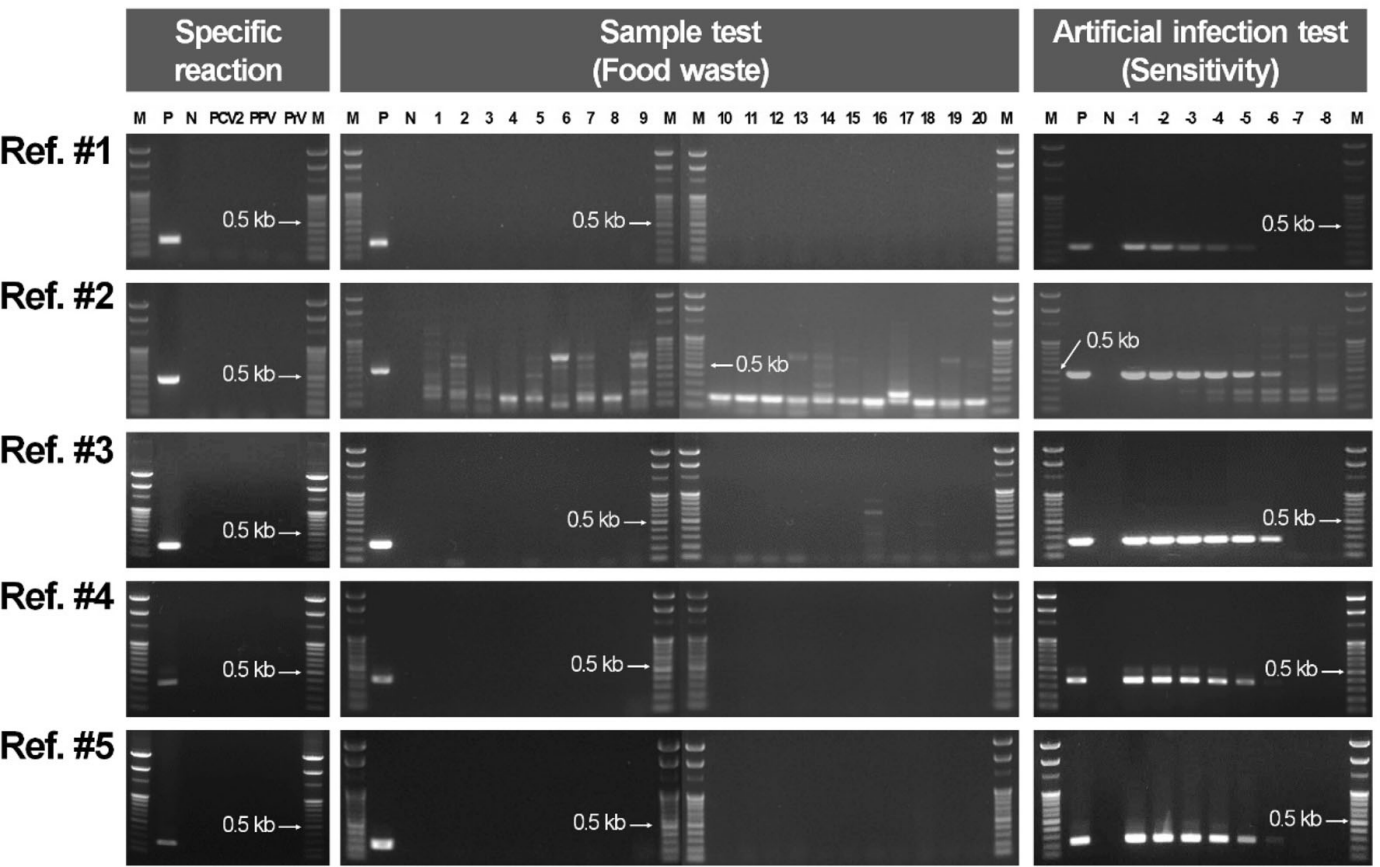
Comparison of specific and non-specific reactions for the twenty food waste samples and the artificial infections based on the sensitivity tests for the five reference PCR primer sets used for the detection of *African swine fever virus* (ASFV). M, 100 bp DNA ladder marker (Enzynomics); P, positive control; N, negative control; PCV2, *porcine circovirus* 2; PPV, *porcine parvovirus*; PrV, *pseudorabies virus*; 1–20, number of food waste samples; –1 to –8; concentration of ASFV nucleic acid artificial infection samples [6, 9, 12–14]

The sensitivity of five conventional PCR primer sets that detect ASFV, including that of Weber et al. [6], were tested for specific and non-specific reactions with food waste samples and artificial infection solutions. The results showed specific reactions with the ASFV plasmid for all five conventional PCR primer sets, and no non-specific reactions were observed with the DNA of the three reference viruses. The results for total DNA extracted from the food waste samples were all negative, but multiple non-specific reactions appeared in reference method #2; thus, the result could not be interpreted. There was also a weak non-specific reaction observed for reference method #3 in the total DNA extracted from the multiple samples. Moreover, the sensitivity for five conventional PCR primer sets with the artificial infection solution was 10^–5^ (10 fg/μL) for reference method #1 and 10^–6^ (1 fg/μL) for methods #2, #3, #4, and #5. In addition, a non-specific reaction was observed in reference method #2 (Fig. 3). The PCR amplicon size and the reaction time for each of the five reference methods were as follows: 257 nt and 77 min for #1; 479 nt and 100 min for #2; 256 nt and 100 min for #3; 310 nt and 70 min for #4; and 243 nt and 70 min for #5, respectively. Among the five reference methods, method #4 was selected for comparison with the developed method because of its high sensitivity in the artificial infection solution with no non-specific reaction to the ASFV-specific reference virus gene and samples. The developed PCR method in this study reacted for 80 min, which is approximately 10 min slower than reference method #4. However, the detection limit in the artificial infection solution was 10^–7^ (100 ag/μL), which was an improvement of approximately 10 times compared to that of the reference method #4. Thus, the developed method could detect ASFV with high sensitivity in food waste samples that may contain PCR inhibitors and fewer copies of targets compared to that of clinical samples. Moreover, the developed method produces 751 nt amplicons, making it more favorable for sequencing, similarity analysis, and genotyping after detection, compared to the shorter 310 nt amplicon formed by reference method #4 (Fig. 3).

### Design and Test of Positive Control Equipped with False-Positive Checking Device

**Fig. 4:**
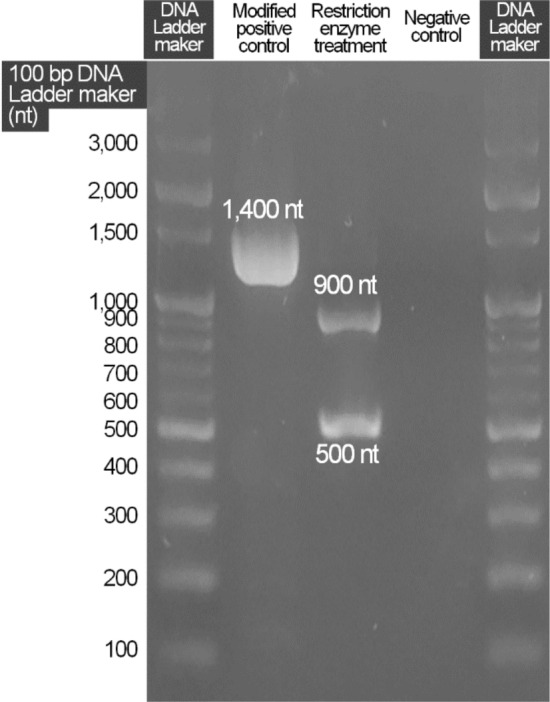
*African swine fever virus* (ASFV) detection using the developed PCR primer set and modified positive controls and the restriction enzyme *Eco*RV digestion 100 bp DNA ladder marker (Enzynomics)

This study developed an ASFV-modified positive material to ensure the accuracy and reliability of the test results. The ASFV primer set #9 can amplify a 751 nt sequence from actual ASFV. However, this primer amplified 1,400 nt sequences in the ASFV-modified positive material. When this PCR product was treated with *Eco*RV, two bands of 500 + 900 nt were identified (Fig. 4 and Supplementary Fig. S1), which enabled the detection of false positives for ASFV diagnosis through gel electrophoresis after PCR. In addition, it can also be used to assess laboratory contamination or to troubleshoot by treatment of the amplified product with *Eco*RV or sequencing for identification using an NCBI basic local alignment search tool (BLAST).

### Validation of the ASFV PCR Diagnostic System

**Fig. 5:**
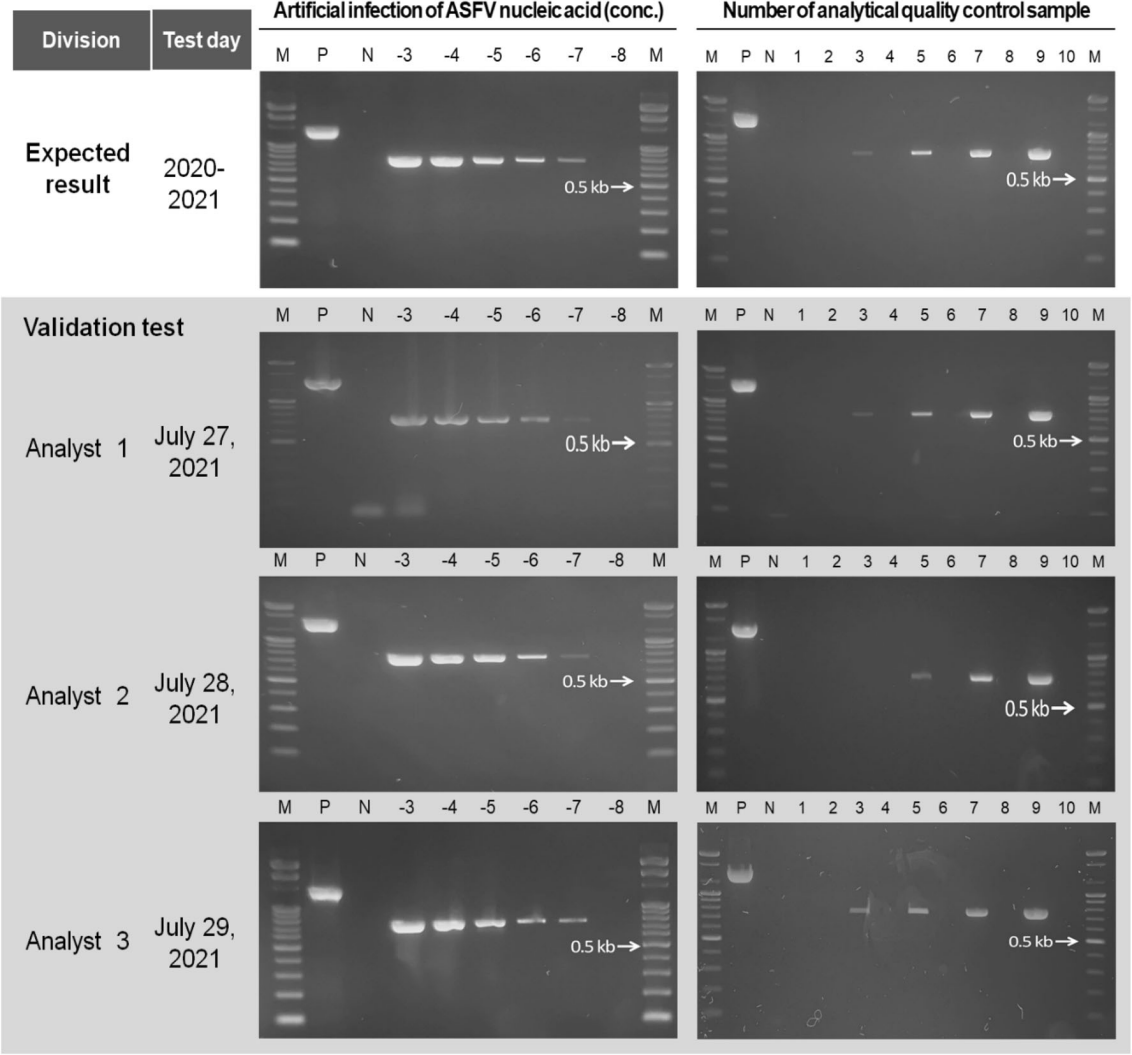
Validation result of developed ASFV PCR diagnostic system in this study based on reproducibility M, 100 bp DNA ladder marker (Enzynomics); P, positive control; N, negative control; -1 ~ -8, dilution value for the template from ASFV plasmid concentration of 1 ng/μL; 1–10, sample number of analytical quality control (AQC) test

The ASFV conventional PCR diagnostic system developed in this investigation was validated by researchers to show its reproducibility, wherein the sensitivity using the artificial infection test was found to be 10^–7^ (100 ag/μL); the AQC for 10 samples was 90% (infected sample with ASFV plasmid 100 ag/μL was not detected) for Researcher 1 and 100% for Researchers 2 and 3 (Fig. 5). PCR analysis was validated based on linearity, accuracy, precision, limit of detection (LOD), and specificity. For linearity, the correlation coefficient, y-axis, the slope of the regression line, residual sum of the square, and correlation coefficient of a straight line based on the readings from the data (> 0.990 is considered excellent) should be analyzed. To prove linearity, the experiment was conducted at five different concentrations, including the target range, and repeated at least three times for each concentration [22]. The results of conventional PCR, unlike those of qPCR where such values can be measured, are interpreted by bands obtained from gel electrophoresis [22]. Hence, here, data for five concentration ranges from three researchers were analyzed in terms of reproducibility for linearity validation and yielded the same results, satisfying linearity. Accuracy was evaluated using results of three repeated measurements (over 2 different days) of samples at four different concentrations, and the unit of accuracy was recovery (%) [22]. Here, ASFV positive samples at different concentrations for 4 out of 10 samples were created to conduct an experiment for testing accuracy, and the three researchers conducted their analyses on different days to test the reproducibility. The average recovery rate among the three researchers was 96.7%. According to the United States Food and Drug Administration (US FDA), LOD refers to the lower limit of quantitation (LLOQ) that satisfies accuracy and precision; and LLOQ is mathematically a concentration that is approximately 10 times the LOD [23]. The ASFV conventional PCR diagnostic system (composition and condition including primer set) developed in this study has an LOD of 100 ag/μL in the food waste sample for the ASFV DNA concentration of (1,422 nt) 1 ng/μL, and the LLOQ was 1 fg/μL. Precision refers to the closeness of the measured values obtained from repeated experiments using a homogenized sample and can be categorized as repeatability (intra-assay precision), intermediate precision, and reproducibility. Generally, when intermediate precision is assessed, the reproducibility is not evaluated [22]. In this study, precision was evaluated based on the reproducibility. The linearity of the qualitative test method and LOD were the same or within the yield range of 80.0–120.0%, confirming the precision of the ASFV conventional PCR diagnostic system developed in this study. To confirm the specificity for validation purposes, the artificial infection test demonstrated that the level of interference was low and that the ASFV-specific amplification could still be performed, although the unknown nucleic acids, inhibitors, resolvents, and PCR buffers were mixed with the samples. Therefore, the ASFV PCR primer set developed in this study was evaluated as an outstanding PCR primer set for the detection of the ASFV gene in food waste with specificity, accuracy, and high sensitivity.

### Application of the Conventional ASFV PCR Diagnostic System with Food Waste Samples

**Fig. 6:**
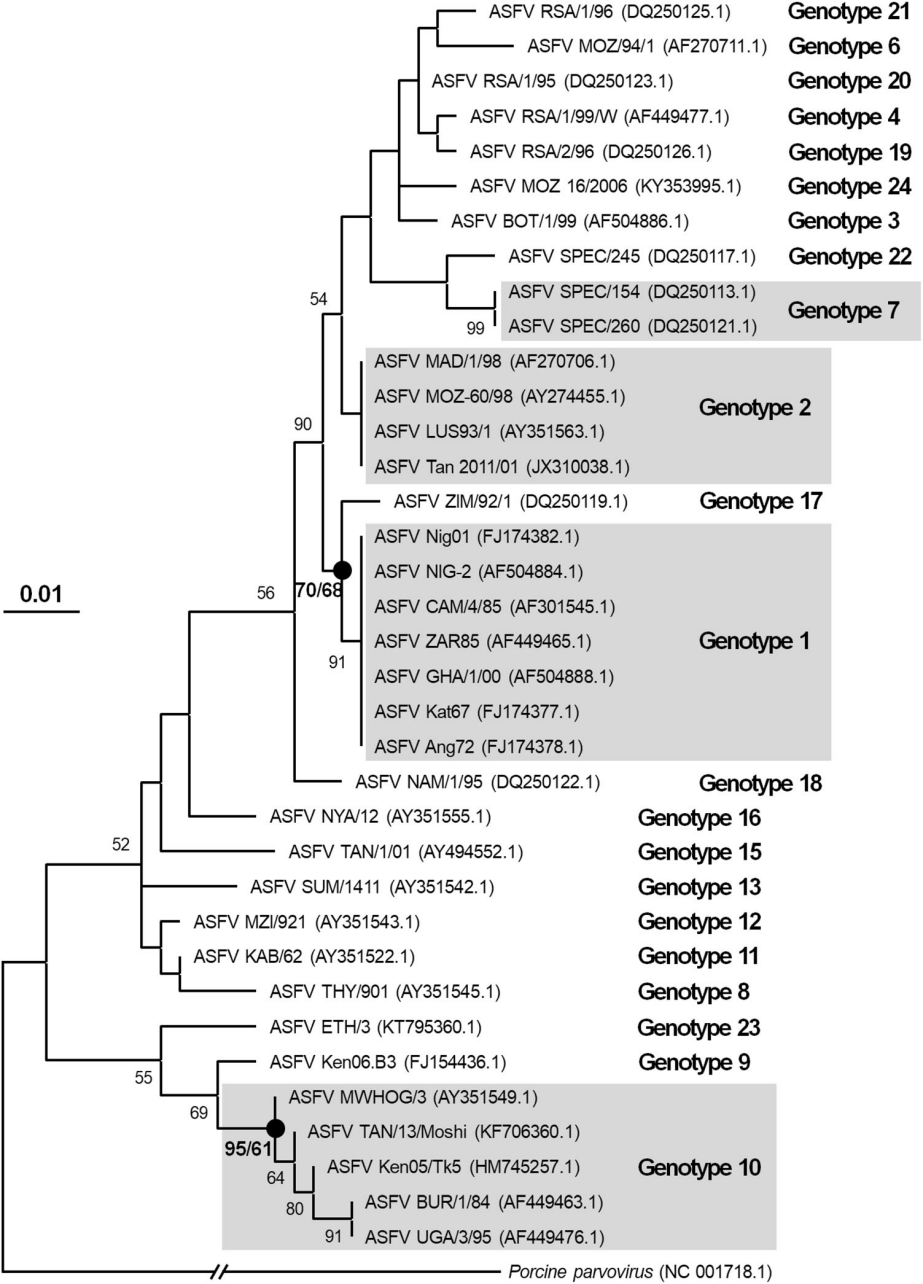
Maximum likelihood (ML) phylogenetic tree showing the phylogenetic position of twenty-four ASFV genotypes based on P72-73 partial gene Evolutionary distances were computed using the Tamura-Nei model. *Porcine parvovirus* (NC 001,718.1) was used as an out-group. Bootstrap values (more than 50%) based on 1,000 replications are shown. Closed circle represents that the corresponding branches were also recovered in bootstrap values from ML/neighbor-joining trees. Bar, 0.01 substitutions per nucleotide position. A broken line is used in the part connected to the out group. However, the branch length is not down to scale

This study developed a diagnostic system that can detect ASFV-specific DNA from food waste at a higher sensitivity when compared to reference PCR methods, with the compositions containing a PCR primer set, PCR conditions, and modified positive material. The diagnostic procedures were as follows: (i) Pork meat-based food waste samples were collected from locations such as households, cafeterias, and treatment facilities, and transported to our laboratory in an icebox. (ii) The samples were ground for 40 s at 6 m/s using the MP FastPrep® 24 (MP Biomedicals) as a pretreatment process (the yield became similar from 40 s [data not shown]). (iii) Total DNA was extracted from 140 μL of the ground solution using a QIAamp® DNA Mini kit (Qiagen), and its concentration measured using a nanodrop. In this study, an average concentration of 185.58 ng/μL (7.53–295.66 ng/μL) and a purity of 1.93 (A260/280; 1.36–2.24) were determined from twenty food waste samples, which were assumed to be suitable for subsequent analysis. (iv) ASFV-specific loci were amplified from the total DNA using the developed method (by ASFV_634F and ASFV_1384R [751 nt] and the modified positive material as a positive control). (v) If the size of the resultant band was approximately 751 nt, it was interpreted as a ‘suspected positive’ and sequencing was performed. When 1400 nt products were indicated, these were the modified positive material indicating that contamination was present, and that troubleshooting should be performed to discover the cause of contamination. In addition, if the amplified PCR product was digested into two bands after treatment with *Eco*RV, the PCR product was confirmed to be from the modified positive material and contamination had certainly occurred. Even if there was contamination from the modified positive material, 751 nt bands from gel electrophoresis could be used for gel purification and sequencing. (vi) Only reliable sequences from the quality graph of the sequencing data were used as query sequences with NCBI BLAST to check for similarity. (vii) Confirmed sequences from sequencing were used for phylogenetic analysis and genotyping. The multiple sequence alignment of the ASFV out-group and reference sequences collected from NCBI with detected sample sequences was conducted using alignment software packages such as BioEdit [24]. Software such as that for molecular evolutionary genetics analysis [25] was used to construct a phylogenetic tree using a maximum likelihood or neighbor-joining based tree making method. The topology of the constructed tree was examined and genotyping of the ASFV sequence from the sample was performed (Fig. 6). Finally, the ASFV was “confirmed” and “identified” with the NCBI BLAST-based similarity and genotyping as evidence.

In contrast, the three real-time PCR Kits (VetMAX™ African Swine Fever Virus Detection Kit [Thermo Fisher Scientific, USA], ID Gene African Swine Fever Duplex [IDVET, France], and POBGEN™ African Swine Fever Virus Detection qPCR kit [PostBio, Korea]), which are commercially available for ASFV diagnosis, had reaction times of 40.0–62.5 min. These were similar to or 40 min faster than reaction times for conventional PCR methods [2]. The sensitivity in food waste samples was 10^–5^ (10 fg/μL)–10^–6^ (1 fg/μL) with a threshold line of 1,000 relative fluorescence units (RFU) as the standard [2]. However, the developed method in this study demonstrated a 10–100 times better LOD than the commercial qPCR kits for ASFV diagnosis, indicating that the developed method is relatively superior for use in food-waste samples (Table 2). Therefore, it is expected that the ASFV diagnostic system developed in this study can be used in conjunction with or independently of qPCR methods. However, it is believed that the p54 gene target, multiplex containing ASFV, and probe modification-based kits that have been recently reported–apart from the kits that are already available commercially–should also be compared to qPCR [26–28] and real-time loop-mediated isothermal amplification methods [29].

**Table 2:** Comparison of evaluation of food waste applicability of PCR methods for *African swine fever virus* diagnosis

**Division**		**Applicability Evaluation in food waste**		**Reaction time (min)**	**Relative cost (Korea won)**	**Reference**
		**Sample test**	**Artificial infection based sensitivity**			
This study	Conventional PCR	Negative	10^-7^ (100 ag/μL)	80.0	Cheap (Approximately 87,000)	This study
Ref. PCR primer set #1		Negative	10^-5^ (10 fg/μL)	77.0		
Ref. PCR primer set #2		Negative (Strong non-specific bands formed)	10^-6^ (1 fg/μL) (Strong non-specific bands formed)	100.0		
Ref. PCR primer set #3		Negative (Weak non-specific bands formed)	10^-6^ (1 fg/μL)	100.0		
Ref. PCR primer set #4		Negative	10^-6^ (1 fg/μL)	70.0		
Ref. PCR primer set #5		Negative	10^-6^ (1 fg/μL)	70.0		
Etc. #1	Quantitative real-time PCR (kit)	Negative	10^-6^ (1 fg/μL)^a^	62.5	Expensive (990,000)^b^	[2]
Etc. #2		Negative	10^-6^ (1 fg/μL)^a^	60.0	Expensive (1,430,000)^b^	
Etc. #3		Negative	10^-5^ (10 fg/μL)^a^	40.0		

^a^This result is the result value shown based on the threshold line 1000 relative fluorescence unit (RFU).

^b^^b^Shows the consumer prices sold in 2021

The cost of the ASFV qPCR kit (based on 96–100 tests) is approximately 880,000–1,540,000 won (average 1,320,000 won) as of 2021 and requires a high-priced machine; some kits require a special equipment [2]. However, the cost of the ASFV diagnostic system developed in this study is approximately 87,000 won (67,000 won for the PCR kit and 20,000 won for the primer based on same tests) (Table 2). Therefore, the same tests can be performed at a cost that is 15 times less expensive, and its performance has been proven to be equivalent to or better than those of other kits. Moreover, as genotyping of relatively long base sequences has become possible, it can be helpful in establishing future response measures–such as those related to epidemiological investigation when ASFV is detected in food waste–as well as offering academic benefits. In 2020, 1516 samples from cafeterias and food waste treatment facilities were monitored for ASFV, and all were found to be negative [2]. However, because there is global concern regarding the possibility of ASFV in food waste, continuous monitoring of ASFV in samples from cafeterias and food-waste treatment facilities (for producing feed and composting) is required to control the potential sources of ASFV and establish scientific evidence for proactive approach. If follow-up studies are conducted on non-specific amplification that cannot be sufficiently identified by monitoring ASFV in food waste samples, then the method developed here can be applied to monitor, identify, and genotyping ASFV in food waste samples.

## Conclusion

ASFV may be present in undercooked meat, as well as in live pigs, and may also cause re-infection or secondary infection from household waste and treatment facilities. Therefore, it is necessary to monitor ASFV in food waste. In this study, we developed a conventional PCR-based diagnostic system that can detect ASFV from various types of food waste samples with high sensitivity. The technique developed could detect nucleic acid fragments from ASFV in food waste at LOD values 10–100 times greater than those of conventional and qPCR-based methods. By using a conventional PCR-based diagnostic technique, testing was possible at a cost approximately 15 times lower than that of qPCR, while sequencing, identifying, and genotyping of relatively long amplified bases sequences was also possible. Therefore, it can be easy to establish government response measures to future outbreaks, such as epidemiological investigation, and may be helpful in providing basic academic data related to ASFV. In addition, we also developed a positive control that can react with the ASFV conventional PCR primer set developed in this study. In the reaction using the positive control developed, a contamination could be classified by using a restriction enzyme fragment length polymorphism (RFLP). It is expected that the technology developed in this study could be applicable to monitoring, identifying, and genotyping ASFV in food waste in the future.

## Supplementary Information

Below is the link to the electronic supplementary material.

Supplementary file1 (PPTX 6932 KB)

Supplementary file2 (PDF 209 KB)

## References

[1] Penrith ML (2020). Current status of African swine fever. CABI Agric Biosci.

[2] National Institute of Environmental Research (2020). Research of food waste characteristics and viral analysis from facilities related to the source of ASF.

[3] Gifford-Gonzalez D, Hanotte O (2011). Domesticating animals in Africa: Implications of genetic and archaeological findings. J World Prehist.

[4] Costard S, Mur L, Lubroth J, Sanchez-Vizcaino JM, Pfeiffer DU (2013). Epidemiology of African swine fever virus. Virus Res.

[5] Michaud V, Randriamparany T, Albina E (2013). Comprehensive phylogenetic reconstructions of African swine fever virus: proposal for a new classification and molecular dating of the virus. PLoS One.

[6] Weber S, Hakobyan A, Zakaryan H, Doerfler W (2018). Intracellular African swine fever virus DNA remains unmethylated in infected Vero cells. Epigenomics.

[7] Borca MV, Ramirez-Medina E, Silva E, Vuono E, Rai A, Pruitt S, Holinka LG, Velazquez-Salinas L, Zhu J, Gladue DP (2020). Development of a highly effective African swine fever virus vaccine by deletion of the I177L gene results in sterile immunity against the current epidemic Eurasia strain. J Virol.

[8] Dixon LK, Stahl K, Jori F, Vial L, Pfeiffer DU (2020). African swine fever epidemiology and control. Annu Rev Anim Biosci.

[9] Patrick BN, Machuka EM, Githae D, Banswe G, Amimo JO, Ongus JR, Masembe C, Bishop RP, Steinaa L, Djikeng A, Pelle R (2020). Evidence for the presence of African swine fever virus in apparently healthy pigs in the South-Kivu Province of the Democratic Republic of Congo. Vet Microbiol.

[10] Ministry of Agriculture, Food and Rural Affairs (2018) Emergency guidelines for African swine fever (SOP)

[11] Animal and Plant Health Agency, Department for Environment, Food & Rural Affairs, and Lord Gardiner of Kimble, (2018) African swine fever risk reminder. https://www.gov.uk/government/news/african-swine-fever-risk-reminder. Accessed 28 Jul 2021. GOV, UK

[12] Agüero M, Fernández J, Romero L, Sánchez Mascaraque C, Arias M, Sánchez-Vizcaíno JM (2003). Highly sensitive PCR assay for routine diagnosis of African swine fever virus in clinical samples. J Clin Microbiol.

[13] Basto AP, Portugal RS, Nix RJ, Cartaxeiro C, Boinas F, Dixon LK, Leitão A, Martins C (2006). Development of a nested PCR and its internal control for the detection of African swine fever virus (ASFV) in Ornithodoros erraticus. Arch Virol.

[14] O’Donnell V, Holinka LG, Krug PW, Gladue DP, Carlson J, Sanford B, Alfano M, Kramer E, Lu Z, Arzt J, Reese B, Carrillo C, Risatti GR, Borca MV (2015). African swine fever virus Georgia 2007 with a deletion of virulence-associated gene 9GL (B119L), when administered at low doses, leads to virus attenuation in swine and induces an effective protection against homologous challenge. J Virol.

[15] Wang A, Jia R, Liu Y, Zhou J, Qi Y, Chen Y, Liu D, Zhao J, Shi H, Zhang J, Zhang G (2020). Development of a novel quantitative real-time PCR assay with lyophilized powder reagent to detect African swine fever virus in blood samples of domestic pigs in China. Transbound Emerg Dis.

[16] Wang Y, Xu L, Noll L, Stoy C, Porter E, Fu J, Feng Y, Peddireddi L, Liu X, Dodd KA, Jia W, Bai J (2020). Development of a real-time PCR assay for detection of African swine fever virus with an endogenous internal control. Transbound Emerg Dis.

[17] Lin Y, Cao C, Shi W, Huang C, Zeng S, Sun J, Wu J, Hua Q (2020). Development of a triplex real-time PCR assay for detection and differentiation of gene-deleted and wild-type African swine fever virus. J Virol Methods.

[18] Yin D, Geng R, Lv H, Bao C, Shao H, Ye J, Qian K, Qin A (2021). Development of real-time PCR based on *A137R* gene for the detection of African swine fever virus. Front Vet Sci.

[19] Lee S, Bae KS, Lee JY, Joo YL, Kim JH, You KA (2021). Development of molecular diagnostic system with high sensitivity for the detection of human Sapovirus from water environments. Biomed Sci [Lett].

[20] Jung S, Lee DY, Choi W, Kang C, Park YE (2018) Introduction of reference materials for water- and foodborne disease viruses Public health weekly Rep. 11, pp 254–259

[21] National Institute of Environmental Research (2018) Research on early detection and characterization of zoonotic disease in wild mammals. Incheon, Republic of Korea. 10.23000/TRKO201900002409

[22] National Institute of Food and Drug Safety Evaluation (2010). Considerations for validation of biodistribution test methods for gene therapy products using quantitative PCR.

[23] United States Food and Drug Administration (2015) Analytical procedures and methods validation for drugs and biologics guidance for industry

[24] Hall TA (1999). BioEdit: A user-friendly biological sequence alignment editor and analysis program for Windows 95/98/NT. Nucleic Acids Symp Ser.

[25] Tamura K, Stecher G, Peterson D, Filipski A, Kumar S (2013). MEGA6: molecular evolutionary genetics analysis version 6.0. Mol Biol Evol.

[26] Trinh TBN, Truong T, Nguyen VT, Vu XD, Dao LA, Nguyen TL, Ambagala A, Babiuk S, Oh J, Song D, Le VP (2021). Development of a novel real-time PCR assay targeting p54 gene for rapid detection of African swine fever virus (ASFV) strains circulating in Vietnam. Vet Med Sci.

[27] Chen Y, Shi K, Liu H, Yin Y, Zhao J, Long F, Lu W, Si H (2021). Development of a multiplex qRT-PCR assay for detection of African swine fever virus, classical swine fever virus and porcine reproductive and respiratory syndrome virus. J Vet Sci.

[28] Tran HTT, Dang AK, Ly DV, Vu HT, Hoang TV, Nguyen CT, Chu NT, Nguyen VT, Nguyen HT, Truong AD, Pham NT, Dang HV (2020). An improvement of real-time polymerase chain reaction system based on probe modification is required for accurate detection of African swine fever virus in clinical samples in Vietnam. Asian-Australas J Anim Sci.

[29] Wang D, Yu J, Wang Y, Zhang M, Li P, Liu M, Liu Y (2019). Development of a real-time loop-mediated isothermal amplification (LAMP) assay and visual LAMP assay for detection of African swine fever virus (ASFV). J Virol Methods.

