# Supplementary material for: Development of an Accurate and Sensitive Diagnostic System Based on Conventional PCR for Detection of African Swine Fever Virus in Food Waste
Source: Indian J Microbiol. 2022 Mar 18;62(2):293–306. doi: 10.1007/s12088-022-01007-y (PMC8980174; doi:10.1007/s12088-022-01007-y)
Supplement: Supplementary file 1 — Supplementary file1 (PPTX 6932 KB) [file 12088_2022_1007_MOESM1_ESM.pptx]

## Slide 1
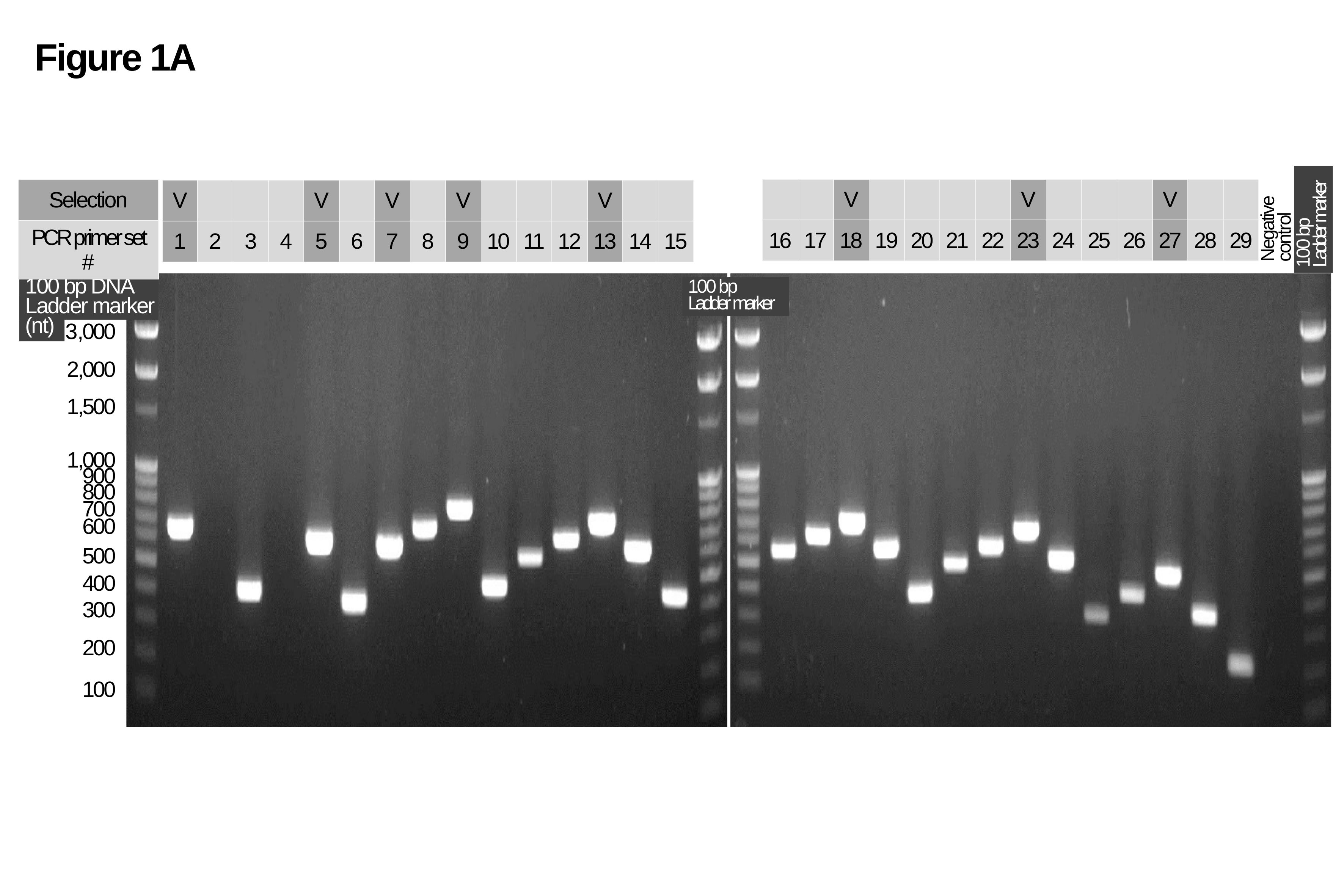

Figure 1A
| | | V | | | | | V | | | | V | | |
| --- | --- | --- | --- | --- | --- | --- | --- | --- | --- | --- | --- | --- | --- |
| 16 | 17 | 18 | 19 | 20 | 21 | 22 | 23 | 24 | 25 | 26 | 27 | 28 | 29 |
| Selection |
| --- |
| PCR primer set # |
| V | | | | V | | V | | V | | | | V | | |
| --- | --- | --- | --- | --- | --- | --- | --- | --- | --- | --- | --- | --- | --- | --- |
| 1 | 2 | 3 | 4 | 5 | 6 | 7 | 8 | 9 | 10 | 11 | 12 | 13 | 14 | 15 |
100 bp
Ladder marker
Negative control
100 bp DNA
Ladder marker
(nt)
100 bp
Ladder marker
3,000
2,000
1,500
1,000
900
800
700
600
500
400
300
200
100

## Slide 2
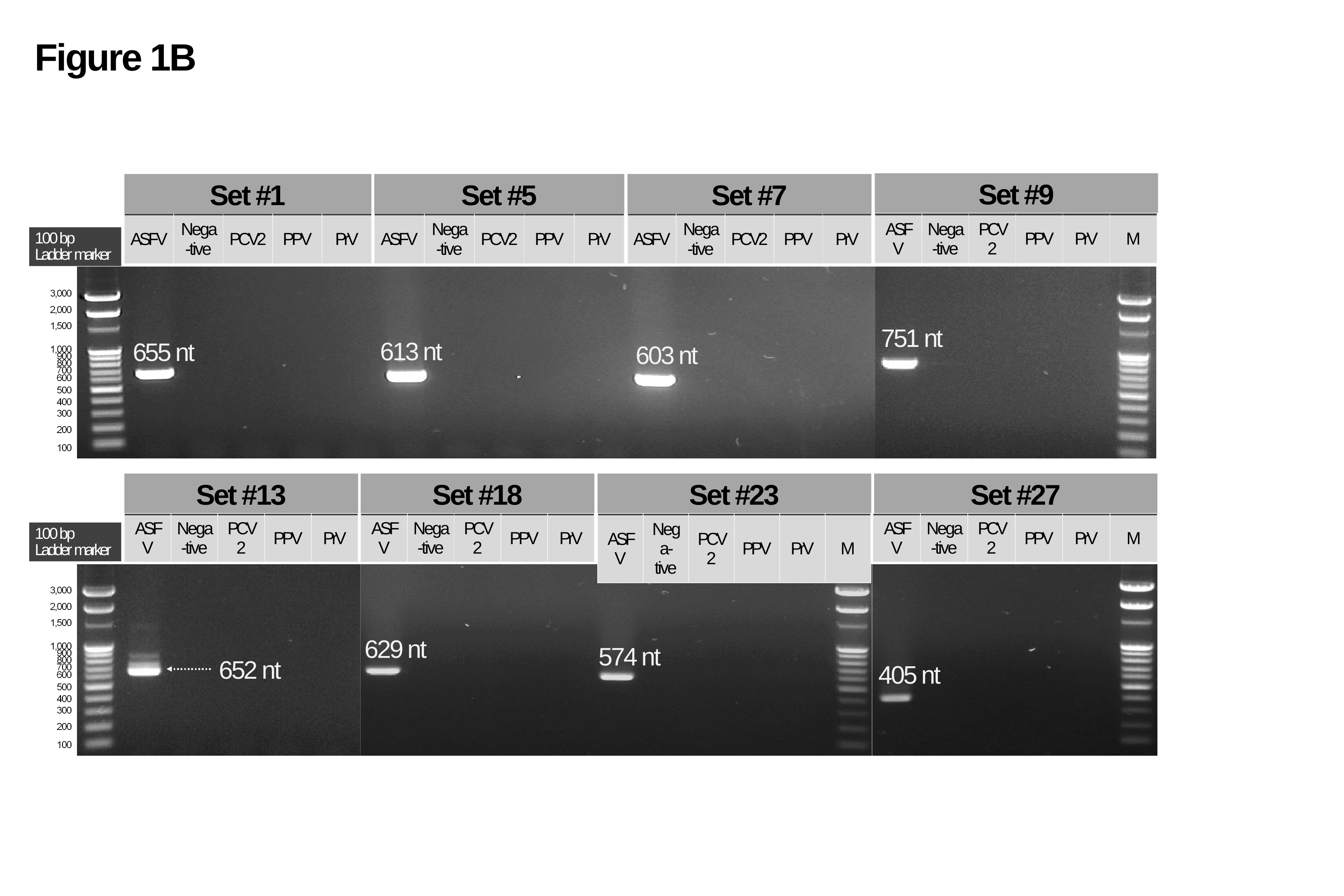

Figure 1B
Set #9
Set #1
Set #5
Set #7
| ASFV | Nega-tive | PCV2 | PPV | PrV | M |
| --- | --- | --- | --- | --- | --- |
| ASFV | Nega-tive | PCV2 | PPV | PrV |
| --- | --- | --- | --- | --- |
| ASFV | Nega-tive | PCV2 | PPV | PrV |
| --- | --- | --- | --- | --- |
| ASFV | Nega-tive | PCV2 | PPV | PrV |
| --- | --- | --- | --- | --- |
100 bp
Ladder marker
751 nt
613 nt
655 nt
603 nt
Set #13
Set #18
Set #23
Set #27
| ASFV | Nega-tive | PCV2 | PPV | PrV |
| --- | --- | --- | --- | --- |
| ASFV | Nega-tive | PCV2 | PPV | PrV |
| --- | --- | --- | --- | --- |
| ASFV | Nega-tive | PCV2 | PPV | PrV | M |
| --- | --- | --- | --- | --- | --- |
| ASFV | Nega-tive | PCV2 | PPV | PrV | M |
| --- | --- | --- | --- | --- | --- |
100 bp
Ladder marker
629 nt
574 nt
652 nt
405 nt

## Slide 3
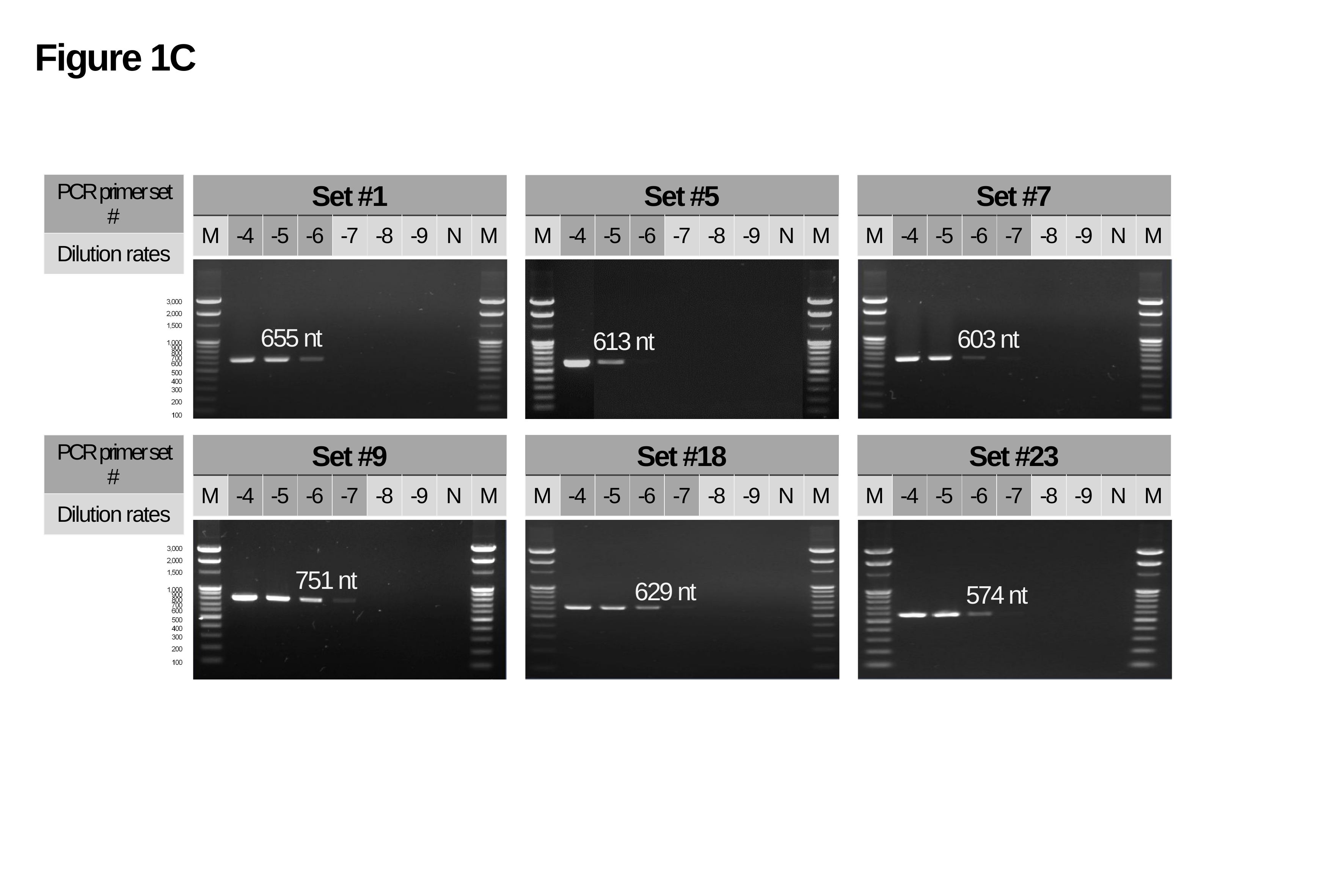

Figure 1C
| PCR primer set # |
| --- |
| Dilution rates |
Set #1
Set #5
Set #7
| M | -4 | -5 | -6 | -7 | -8 | -9 | N | M |
| --- | --- | --- | --- | --- | --- | --- | --- | --- |
| M | -4 | -5 | -6 | -7 | -8 | -9 | N | M |
| --- | --- | --- | --- | --- | --- | --- | --- | --- |
| M | -4 | -5 | -6 | -7 | -8 | -9 | N | M |
| --- | --- | --- | --- | --- | --- | --- | --- | --- |
655 nt
603 nt
613 nt
| PCR primer set # |
| --- |
| Dilution rates |
Set #9
Set #18
Set #23
| M | -4 | -5 | -6 | -7 | -8 | -9 | N | M |
| --- | --- | --- | --- | --- | --- | --- | --- | --- |
| M | -4 | -5 | -6 | -7 | -8 | -9 | N | M |
| --- | --- | --- | --- | --- | --- | --- | --- | --- |
| M | -4 | -5 | -6 | -7 | -8 | -9 | N | M |
| --- | --- | --- | --- | --- | --- | --- | --- | --- |
751 nt
629 nt
574 nt

## Slide 4
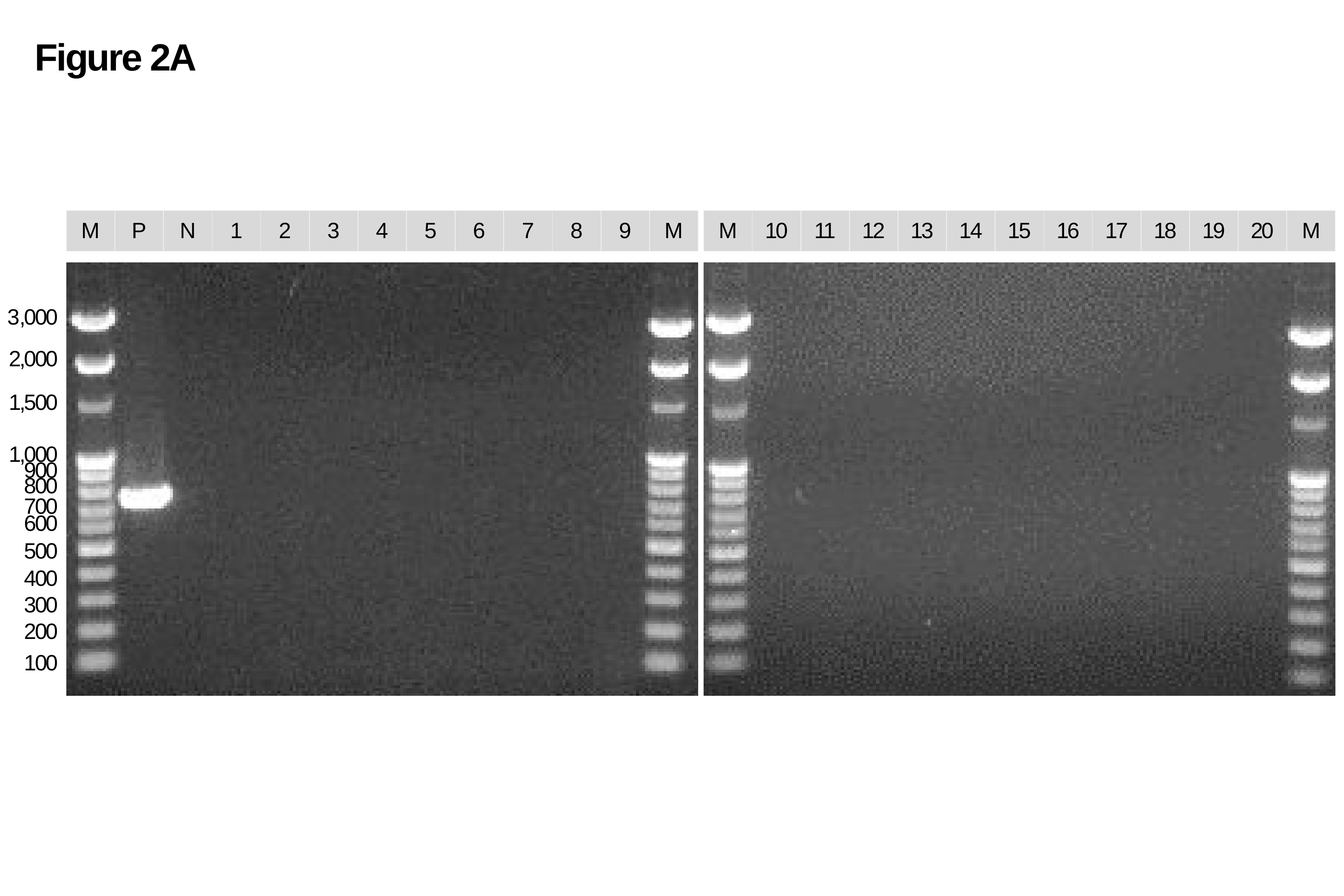

| Set #9 | | | | | | | | | | | |
| --- | --- | --- | --- | --- | --- | --- | --- | --- | --- | --- | --- |
| M | P | N | -1 | -2 | -3 | -4 | -5 | -6 | -7 | -8 | M |
Figure 2A
| M | P | N | 1 | 2 | 3 | 4 | 5 | 6 | 7 | 8 | 9 | M |
| --- | --- | --- | --- | --- | --- | --- | --- | --- | --- | --- | --- | --- |
| M | 10 | 11 | 12 | 13 | 14 | 15 | 16 | 17 | 18 | 19 | 20 | M |
| --- | --- | --- | --- | --- | --- | --- | --- | --- | --- | --- | --- | --- |
3,000
2,000
1,500
1,000
900
800
700
600
500
400
300
200
100

## Slide 5
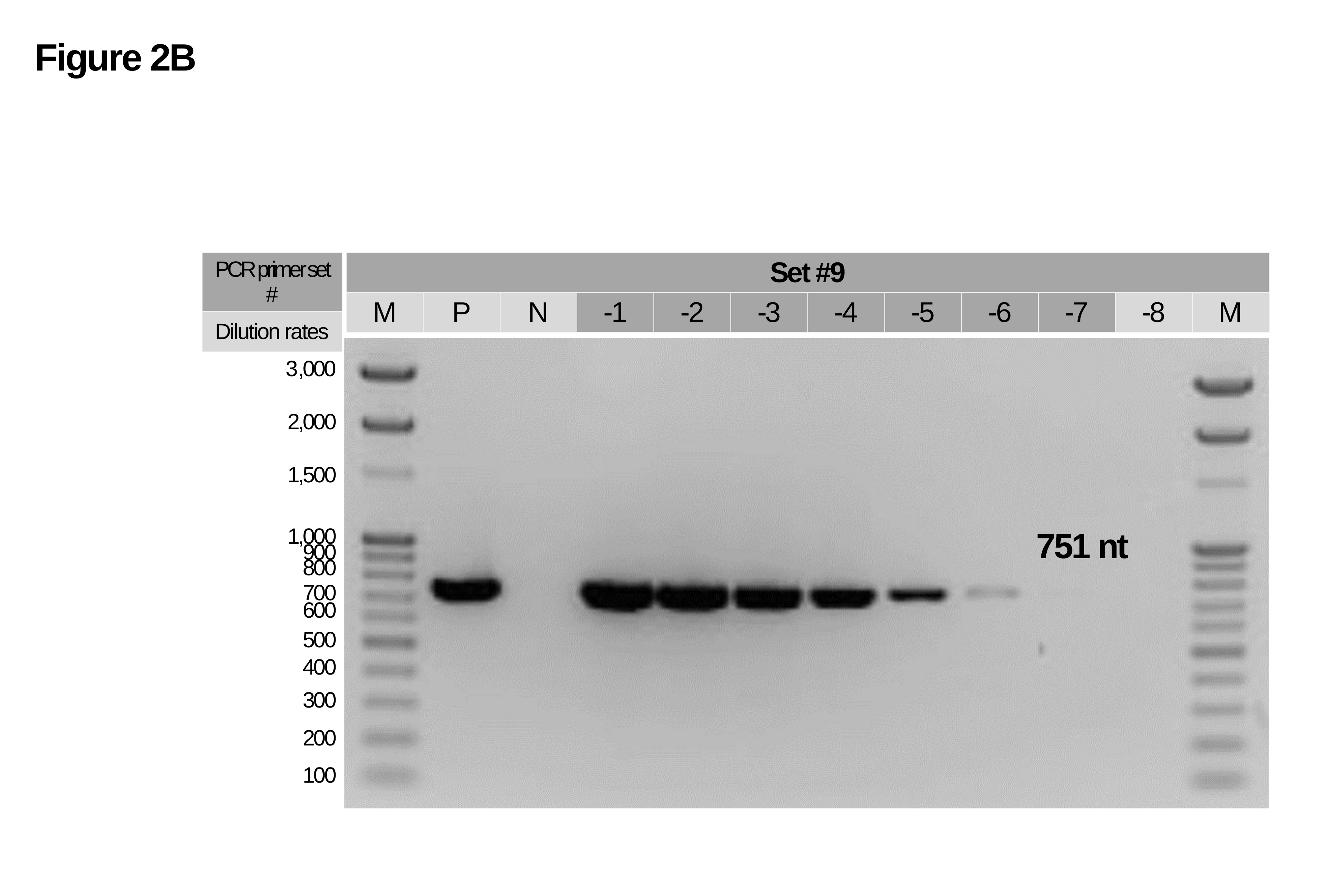

Figure 2B
| PCR primer set # |
| --- |
| Dilution rates |
| Set #9 | | | | | | | | | | | |
| --- | --- | --- | --- | --- | --- | --- | --- | --- | --- | --- | --- |
| M | P | N | -1 | -2 | -3 | -4 | -5 | -6 | -7 | -8 | M |
3,000
2,000
1,500
1,000
900
800
700
600
500
400
300
200
100
751 nt

## Slide 6
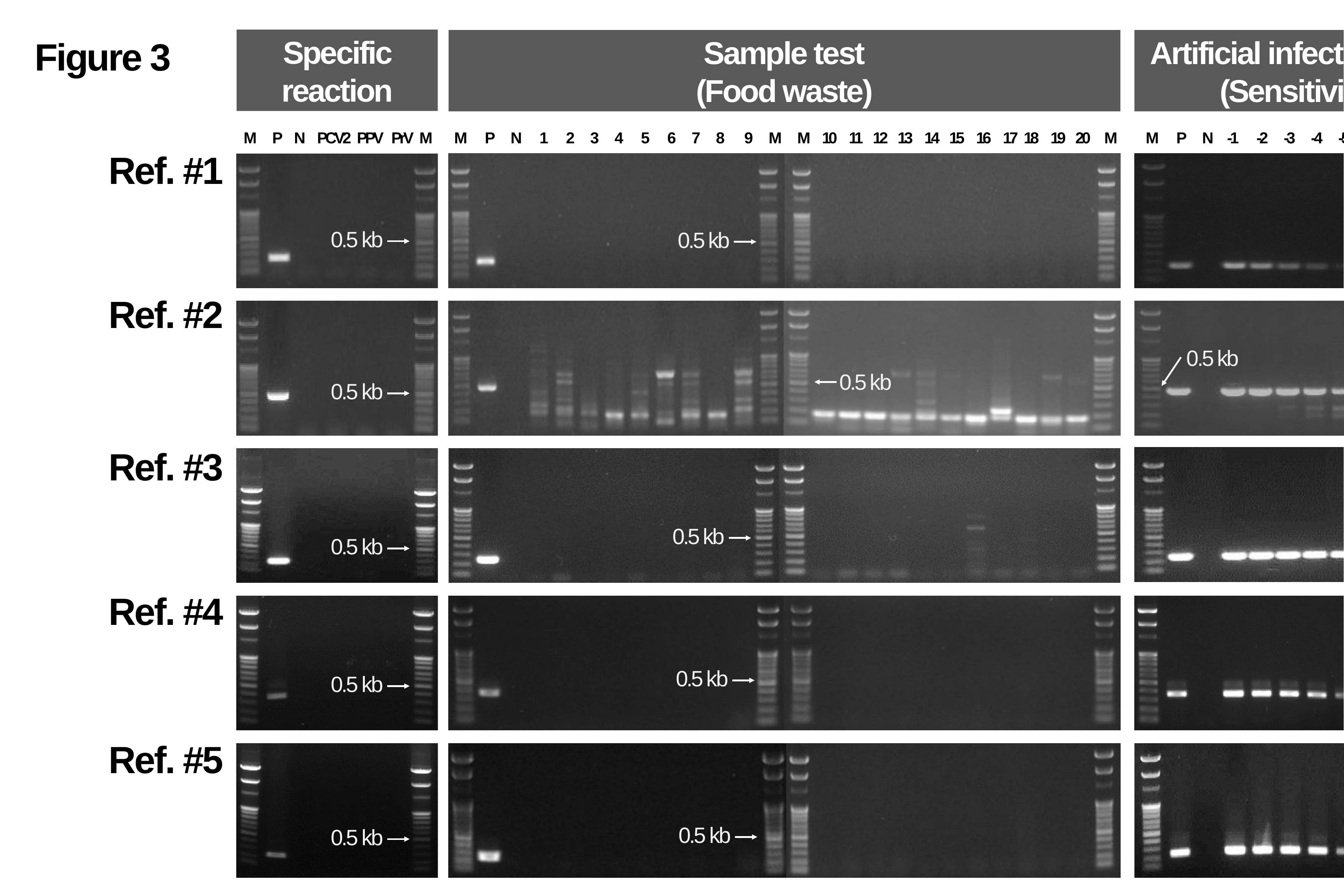

Specific reaction
Artificial infection test (Sensitivity)
Figure 3
Sample test
(Food waste)
M P N PCV2 PPV PrV M
M P N -1 -2 -3 -4 -5 -6 -7 -8 M
M P N 1 2 3 4 5 6 7 8 9 M M 10 11 12 13 14 15 16 17 18 19 20 M
Ref. #1
0.5 kb
0.5 kb
0.5 kb
Ref. #2
0.5 kb
0.5 kb
0.5 kb
Ref. #3
0.5 kb
0.5 kb
0.5 kb
Ref. #4
0.5 kb
0.5 kb
0.5 kb
Ref. #5
0.5 kb
0.5 kb
0.5 kb

## Slide 7
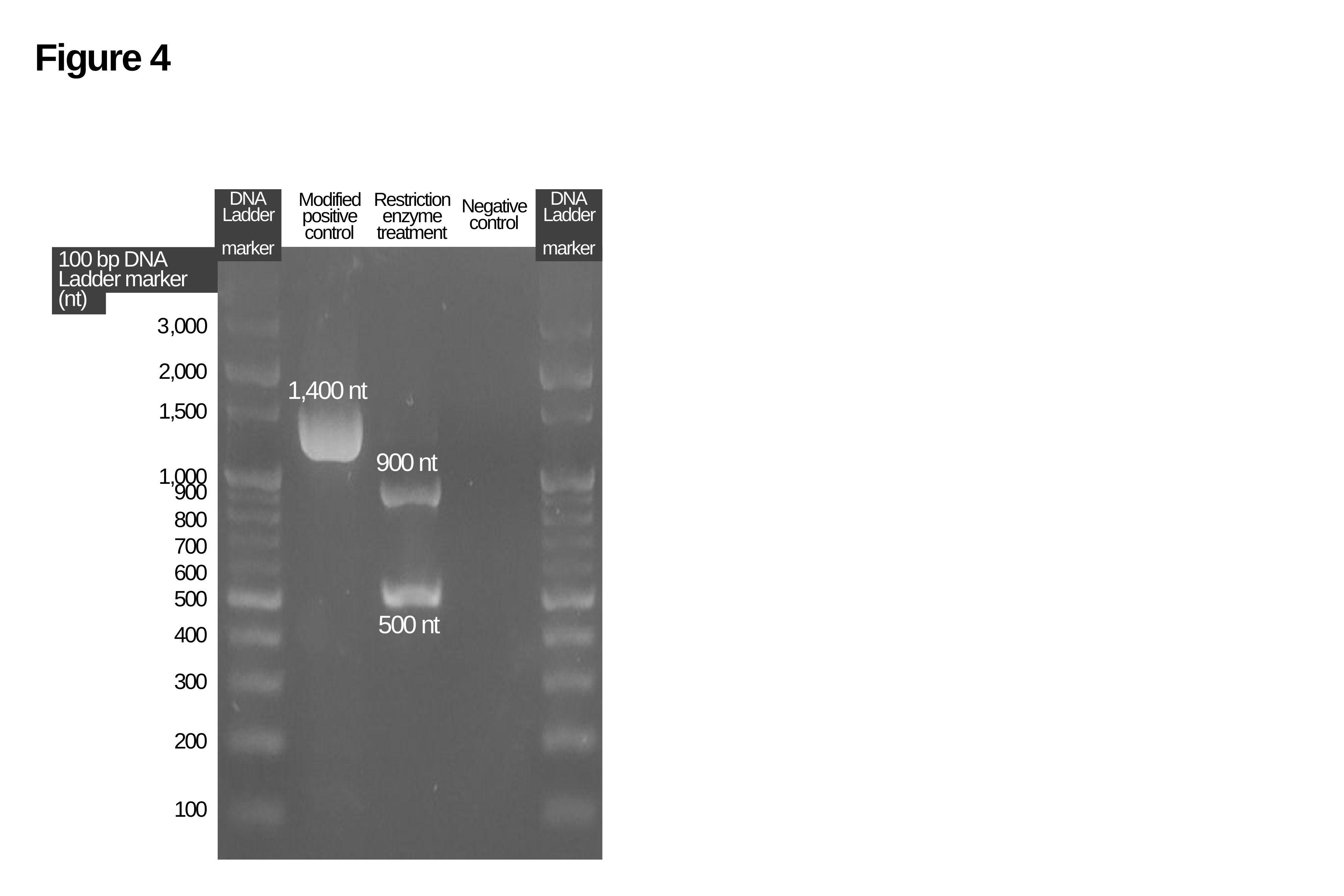

Figure 4
DNA
Ladder
marker
DNA
Ladder
marker
Modified positive control
Restriction enzyme treatment
Negative control
100 bp DNA
Ladder marker
(nt)
3,000
2,000
1,500
1,000
900
800
700
600
500
400
300
200
100
1,400 nt
900 nt
500 nt

## Slide 8
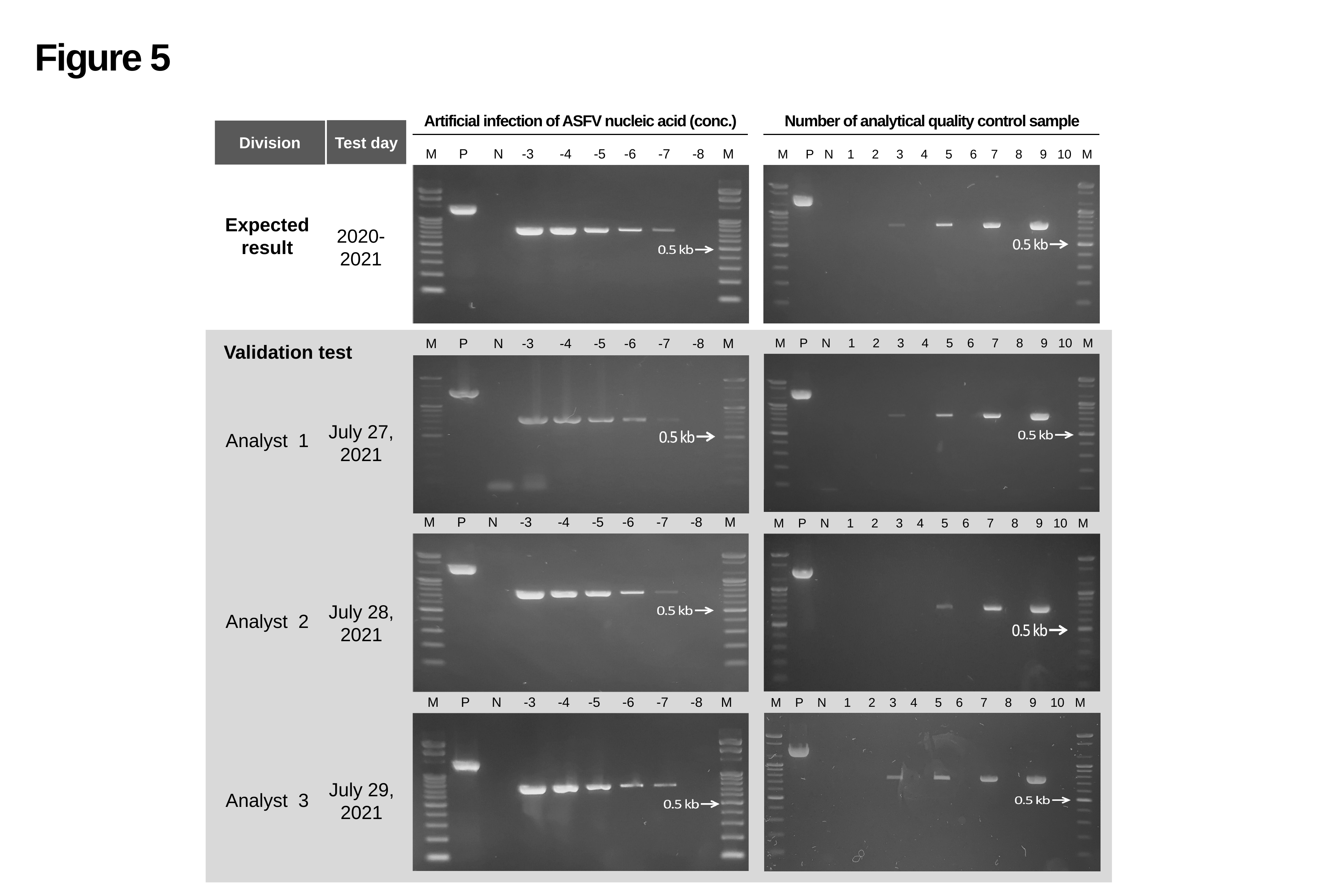

Figure 5
Artificial infection of ASFV nucleic acid (conc.)
Number of analytical quality control sample
Test day
Division
 M P N -3 -4 -5 -6 -7 -8 M
M P N 1 2 3 4 5 6 7 8 9 10 M
Expected result
2020-2021
 M P N -3 -4 -5 -6 -7 -8 M
M P N 1 2 3 4 5 6 7 8 9 10 M
Validation test
July 27,
2021
Analyst 1
 M P N -3 -4 -5 -6 -7 -8 M
 M P N 1 2 3 4 5 6 7 8 9 10 M
July 28,
2021
Analyst 2
 M P N -3 -4 -5 -6 -7 -8 M
M P N 1 2 3 4 5 6 7 8 9 10 M
July 29,
2021
Analyst 3

## Slide 9
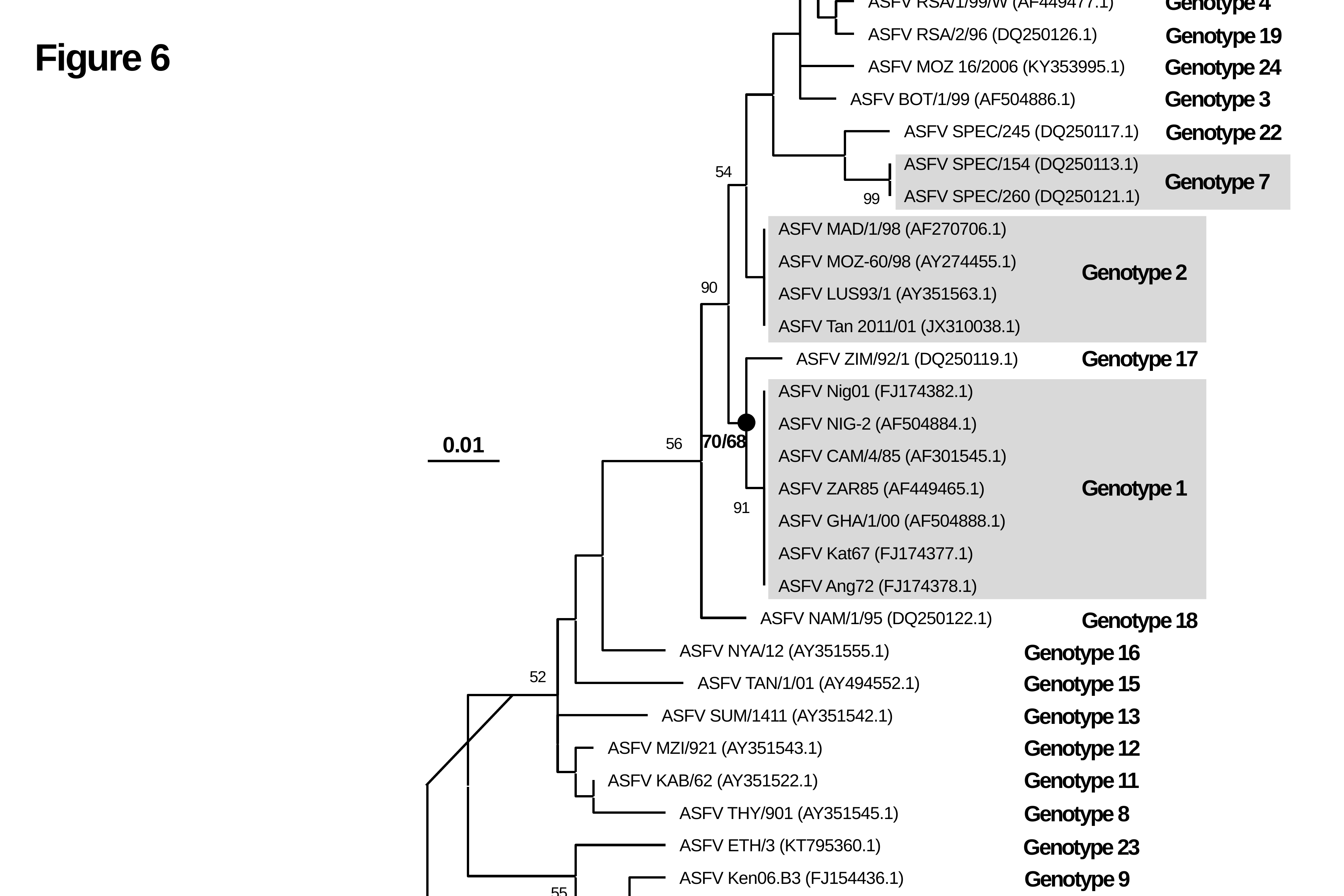

Genotype 21
 ASFV RSA/1/96 (DQ250125.1)
Genotype 6
 ASFV MOZ/94/1 (AF270711.1)
Genotype 20
 ASFV RSA/1/95 (DQ250123.1)
Genotype 4
 ASFV RSA/1/99/W (AF449477.1)
Genotype 19
 ASFV RSA/2/96 (DQ250126.1)
Figure 6
Genotype 24
 ASFV MOZ 16/2006 (KY353995.1)
Genotype 3
 ASFV BOT/1/99 (AF504886.1)
Genotype 22
 ASFV SPEC/245 (DQ250117.1)
 ASFV SPEC/154 (DQ250113.1)
54
Genotype 7
 ASFV SPEC/260 (DQ250121.1)
99
 ASFV MAD/1/98 (AF270706.1)
 ASFV MOZ-60/98 (AY274455.1)
Genotype 2
90
 ASFV LUS93/1 (AY351563.1)
 ASFV Tan 2011/01 (JX310038.1)
Genotype 17
 ASFV ZIM/92/1 (DQ250119.1)
 ASFV Nig01 (FJ174382.1)
 ASFV NIG-2 (AF504884.1)
70/68
0.01
56
 ASFV CAM/4/85 (AF301545.1)
Genotype 1
 ASFV ZAR85 (AF449465.1)
91
 ASFV GHA/1/00 (AF504888.1)
 ASFV Kat67 (FJ174377.1)
 ASFV Ang72 (FJ174378.1)
Genotype 18
 ASFV NAM/1/95 (DQ250122.1)
Genotype 16
 ASFV NYA/12 (AY351555.1)
52
Genotype 15
 ASFV TAN/1/01 (AY494552.1)
Genotype 13
 ASFV SUM/1411 (AY351542.1)
Genotype 12
 ASFV MZI/921 (AY351543.1)
Genotype 11
 ASFV KAB/62 (AY351522.1)
Genotype 8
 ASFV THY/901 (AY351545.1)
Genotype 23
 ASFV ETH/3 (KT795360.1)
Genotype 9
 ASFV Ken06.B3 (FJ154436.1)
55
 ASFV MWHOG/3 (AY351549.1)
69
 ASFV TAN/13/Moshi (KF706360.1)
95/61
Genotype 10
 ASFV Ken05/Tk5 (HM745257.1)
64
 ASFV BUR/1/84 (AF449463.1)
80
 ASFV UGA/3/95 (AF449476.1)
91
 Porcine parvovirus (NC 001718.1)

## Slide 10
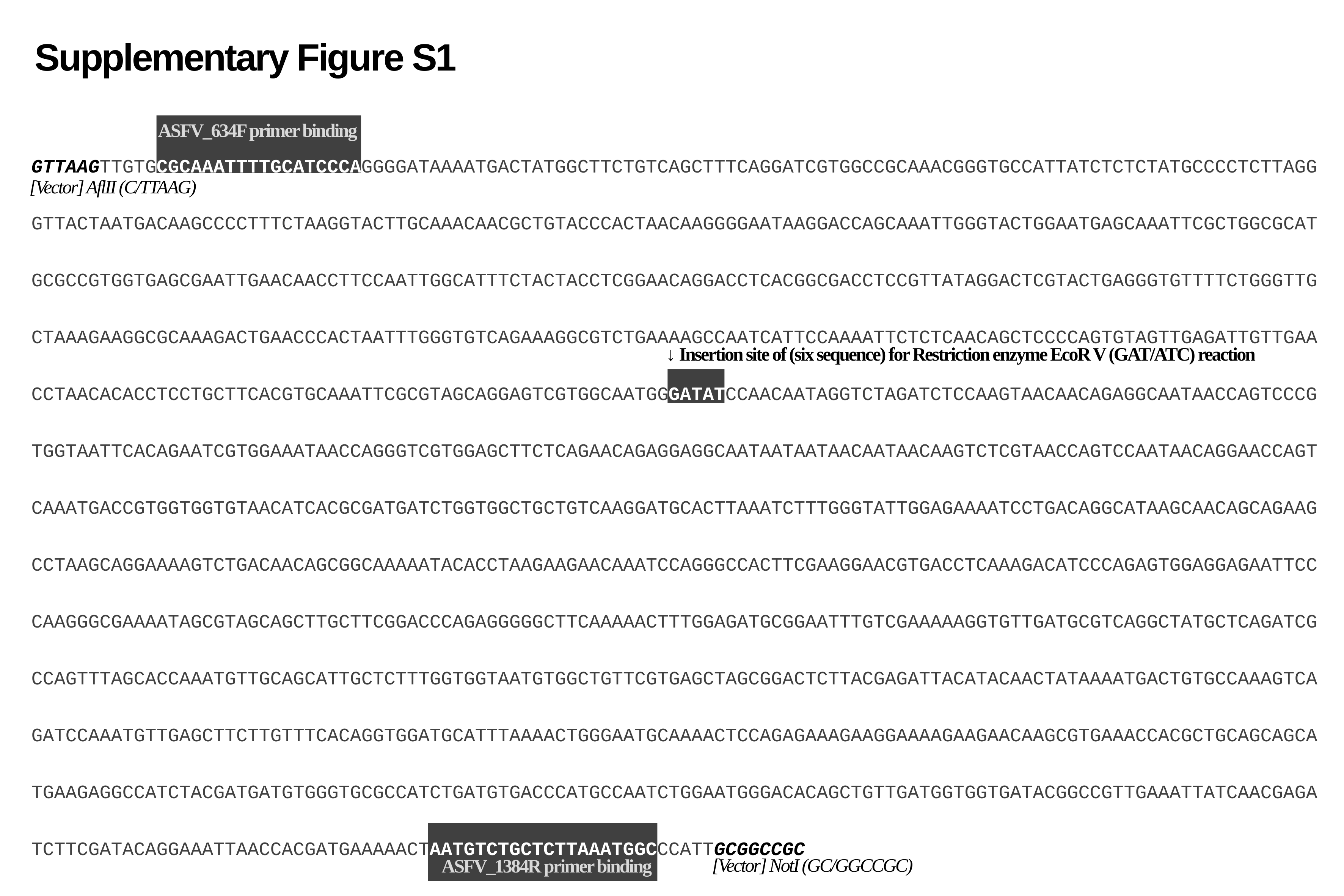

Supplementary Figure S1
ASFV_634F primer binding
GTTAAGTTGTGCGCAAATTTTGCATCCCAGGGGATAAAATGACTATGGCTTCTGTCAGCTTTCAGGATCGTGGCCGCAAACGGGTGCCATTATCTCTCTATGCCCCTCTTAGGGTTACTAATGACAAGCCCCTTTCTAAGGTACTTGCAAACAACGCTGTACCCACTAACAAGGGGAATAAGGACCAGCAAATTGGGTACTGGAATGAGCAAATTCGCTGGCGCATGCGCCGTGGTGAGCGAATTGAACAACCTTCCAATTGGCATTTCTACTACCTCGGAACAGGACCTCACGGCGACCTCCGTTATAGGACTCGTACTGAGGGTGTTTTCTGGGTTGCTAAAGAAGGCGCAAAGACTGAACCCACTAATTTGGGTGTCAGAAAGGCGTCTGAAAAGCCAATCATTCCAAAATTCTCTCAACAGCTCCCCAGTGTAGTTGAGATTGTTGAACCTAACACACCTCCTGCTTCACGTGCAAATTCGCGTAGCAGGAGTCGTGGCAATGGGATATCCAACAATAGGTCTAGATCTCCAAGTAACAACAGAGGCAATAACCAGTCCCGTGGTAATTCACAGAATCGTGGAAATAACCAGGGTCGTGGAGCTTCTCAGAACAGAGGAGGCAATAATAATAACAATAACAAGTCTCGTAACCAGTCCAATAACAGGAACCAGTCAAATGACCGTGGTGGTGTAACATCACGCGATGATCTGGTGGCTGCTGTCAAGGATGCACTTAAATCTTTGGGTATTGGAGAAAATCCTGACAGGCATAAGCAACAGCAGAAGCCTAAGCAGGAAAAGTCTGACAACAGCGGCAAAAATACACCTAAGAAGAACAAATCCAGGGCCACTTCGAAGGAACGTGACCTCAAAGACATCCCAGAGTGGAGGAGAATTCCCAAGGGCGAAAATAGCGTAGCAGCTTGCTTCGGACCCAGAGGGGGCTTCAAAAACTTTGGAGATGCGGAATTTGTCGAAAAAGGTGTTGATGCGTCAGGCTATGCTCAGATCGCCAGTTTAGCACCAAATGTTGCAGCATTGCTCTTTGGTGGTAATGTGGCTGTTCGTGAGCTAGCGGACTCTTACGAGATTACATACAACTATAAAATGACTGTGCCAAAGTCAGATCCAAATGTTGAGCTTCTTGTTTCACAGGTGGATGCATTTAAAACTGGGAATGCAAAACTCCAGAGAAAGAAGGAAAAGAAGAACAAGCGTGAAACCACGCTGCAGCAGCATGAAGAGGCCATCTACGATGATGTGGGTGCGCCATCTGATGTGACCCATGCCAATCTGGAATGGGACACAGCTGTTGATGGTGGTGATACGGCCGTTGAAATTATCAACGAGATCTTCGATACAGGAAATTAACCACGATGAAAAACTAATGTCTGCTCTTAAATGGCCCATTGCGGCCGC
[Vector] AflII (C/TTAAG)
↓ Insertion site of (six sequence) for Restriction enzyme EcoR V (GAT/ATC) reaction
[Vector] NotI (GC/GGCCGC)
ASFV_1384R primer binding
