# Supplementary material for: Development of an Accurate and Sensitive Diagnostic System Based on Conventional PCR for Detection of African Swine Fever Virus in Food Waste
Source: Indian J Microbiol. 2022 Mar 18;62(2):293–306. doi: 10.1007/s12088-022-01007-y (PMC8980174; doi:10.1007/s12088-022-01007-y)
Supplement: Supplementary file 2 — Supplementary file2 (PDF 209 KB) [file 12088_2022_1007_MOESM2_ESM.pdf]

ASFV_634F primer binding

GTTAAGTTGTG**CGCAAATTTTGCATCCCA**GGGGATAAAATGACTATGGCTTCTGTCAGCTTTCAGGATCGTGGCCGCAAACGGGTGCCATTATCTCTCTATGCCCCTCTTAGG

[Vector] AflII (C/TTAAG)

GTTACTAATGACAAGCCCCTTTCTAAGGTACTTGCAAACAACGCTGTACCCACTAACAAGGGGAATAAGGACCAGCAAATTGGGTACTGGAATGAGCAAATTCGCTGGCGCAT

GCGCCGTGGTGAGCGAATTGAACAACCTTCCAATTGGCATTTCTACTACCTCGGAACAGGACCTCACGGCGACCTCCGTTATAGGACTCGTACTGAGGGTGTTTTCTGGGTTG

CTAAAGAAGGCGCAAAGACTGAACCCACTAATTTGGGTGTCAGAAAGGCGTCTGAAAAGCCAATCATTCCAAAATTCTCTCAACAGCTCCCCAGTGTAGTTGAGATTGTTGAA

↓ **Insertion site of (six sequence) for Restriction enzyme EcoR V (GAT/ATC) reaction**

CCTAACACACCTCCTGCTTCACGTGCAAATTCGCGTAGCAGGAGTCGTGGCAATGG**GATAT**CCAACAATAGGTCTAGATCTCCAAGTAACAACAGAGGCAATAACCAGTCCCG

TGGTAATTCACAGAATCGTGGAAATAACCAGGGTCGTGGAGCTTCTCAGAACAGAGGAGGCAATAATAATAACAATAACAAGTCTCGTAACCAGTCCAATAACAGGAACCAGT

CAAATGACCGTGGTGGTGTAACATCACGCGATGATCTGGTGGCTGCTGTCAAGGATGCACTTAAATCTTTGGGTATTGGAGAAAATCCTGACAGGCATAAGCAACAGCAGAAG

CCTAAGCAGGAAAAGTCTGACAACAGCGGCAAAAATACACCTAAGAAGAACAAATCCAGGGCCACTTCGAAGGAACGTGACCTCAAAGACATCCCAGAGTGGAGGAGAATTCC

CAAGGGCGAAAATAGCGTAGCAGCTTGCTTCGGACCCAGAGGGGGCTTCAAAAACTTTGGAGATGCGGAATTTGTCGAAAAAGGTGTTGATGCGTCAGGCTATGCTCAGATCG

CCAGTTTAGCACCAAATGTTGCAGCATTGCTCTTTGGTGGTAATGTGGCTGTTCGTGAGCTAGCGGACTCTTACGAGATTACATACAACTATAAAATGACTGTGCCAAAGTCA

GATCCAAATGTTGAGCTTCTTGTTTCACAGGTGGATGCATTTAAAACTGGGAATGCAAAACTCCAGAGAAAGAAGGAAAAGAAGAACAAGCGTGAAACCACGCTGCAGCAGCA

TGAAGAGGCCATCTACGATGATGTGGGTGCGCCATCTGATGTGACCCATGCCAATCTGGAATGGGACACAGCTGTTGATGGTGGTGATACGGCCGTTGAAATTATCAACGAGA

TCTTCGATACAGGAAATTAACCACGATGAAAAACT**AATGTCTGCTCTTAAATGGC**CCATT***GCGGCCGC***

ASFV_1384R primer binding *[Vector] NotI (GC/GGCCGC)*

Supplementary Fig. S1.
